# Neural Efficiency and Sensorimotor Adaptations in Swimming Athletes: A Systematic Review of Neuroimaging and Cognitive–Behavioral Evidence for Performance and Wellbeing

**DOI:** 10.3390/brainsci16010116

**Published:** 2026-01-22

**Authors:** Evgenia Gkintoni, Andrew Sortwell, Apostolos Vantarakis

**Affiliations:** 1 Lab of Public Health, Epidemiology and Quality of Life, Department of Medicine, University of Patras, 26504 Patras, Greece; 2 Department of Psychiatry, University General Hospital of Patras, 26504 Patras, Greece; 3 School of Education, The University of Notre-Dame Australia, Sydney, NSW 2007, Australia; andrew.sortwell@nd.edu.au; 4 School of Health Sciences and Physiotherapy, University of Notre Dame Australia, 32 Mouat St., Fremantle, WA 6160, Australia; 5 Research Centre in Sports, Health and Human Development, University of Beira Interior, 6201-001 Covilhã, Portugal

**Keywords:** swimming, cognitive performance, neural adaptation, neuroimaging, EEG, fMRI, attention, mental fatigue, motor expertise, brain connectivity, neuroplasticity

## Abstract

**Background/Objectives:** Swimming requires precise motor control, sustained attention, and optimal cognitive–motor integration, making it an ideal model for investigating neural efficiency—the phenomenon whereby expert performers achieve optimal outcomes with reduced neural resource expenditure, operationalized as lower activation, sparser connectivity, and enhanced functional integration. This systematic review examined cognitive performance and neural adaptations in swimming athletes, investigating neuroimaging and behavioral outcomes distinguishing swimmers from non-athletes across performance levels. **Methods:** Following PRISMA 2020 guidelines, seven databases were searched (1999–2024) for studies examining cognitive/neural outcomes in swimmers using neuroimaging or validated assessments. A total of 24 studies (neuroimaging: *n* = 9; behavioral: *n* = 15) met the inclusion criteria. Risk of bias assessment used adapted Cochrane RoB2 and Newcastle–Ottawa Scale criteria. **Results:** Neuroimaging modalities included EEG (*n* = 4), fMRI (*n* = 2), TMS (*n* = 1), and ERP (*n* = 2). Key associations identified included the following: (1) Neural Efficiency: elite swimmers showed sparser upper beta connectivity (35% fewer connections, d = 0.76, *p* = 0.040) and enhanced alpha rhythm intensity (*p* ≤ 0.01); (2) Cognitive Performance: superior attention, working memory, and executive control correlated with expertise (d = 0.69–1.31), with thalamo-sensorimotor functional connectivity explaining 41% of world ranking variance (r^2^ = 0.41, *p* < 0.001); (3) Attention: external focus strategies improved performance in intermediate swimmers but showed inconsistent effects in experts; (4) Mental Fatigue: impaired performance in young adult swimmers (1.2% decrement, d = 0.13) but not master swimmers (*p* = 0.49); (5) Genetics: COMT Val158Met polymorphism associated with performance differences (*p* = 0.026). Effect sizes ranged from small to large, with Cohen’s d = 0.13–1.31. **Conclusions:** Swimming expertise is associated with specific neural and cognitive characteristics, including efficient brain connectivity and enhanced cognitive control. However, cross-sectional designs (88% of studies) and small samples (median *n* = 36; all studies underpowered) preclude causal inference. The lack of spatially quantitative synthesis and visualization of neuroimaging findings represents a methodological limitation of this review and the field. The findings suggest potential applications for talent identification, training optimization, and mental health promotion through swimming but require longitudinal validation and development of standardized swimmer brain atlases before definitive recommendations.

## 1. Introduction

Swimming is one of the most cognitively and physically demanding activities, as it necessitates optimal motor control, sustained attention, and optimal integration of cognition and motor systems in an aqueous environment with demanding environmental conditions [1,2,3,4]. Aquatic sport demands that swimmers process high levels of sensory/motor information and make decisions under conditions that affect all sensory and motor modalities. The relationship between cognitive performance and/or neural adaptation in swimming has been shown to be one of great interest, with various consequences in research characterized by swimming and aquatic performance.

Swimming represents an exemplary model of neuroplasticity study, thanks to its set of characteristics that allow it to be uniquely comprehensive. Firstly, swimming inherently demands a spatial three-dimensional orientation, which goes far beyond what has to be accomplished by ground movement, as one has to watch not only one’s relationship with the water surface, but also with swimming pool boundaries and competition rivals at the same time [5,6]. Secondly, swimming, thanks to its demands of coordinating bilateral movement, temporal movement cycles, and cycle-breathing coordination, represents one of those extraordinary tasks that, at least in its principal form, never has to replicate such high coordination demands through comparable, exclusively ground movement [7,8,9]. Additionally, swimming, due to reduced ground contact feedback, demands and develops high-level predictive motor control and mental representation of body movement [10,11]. Finally, water resistance, buoyancy, and water temperature, as environmental demands, impose continuous sensorimotor adjustments during swimming practice [12].

Recent technological advances in neuroimaging and computational neuroscience provide unparalleled approaches to investigate the neural mechanisms involved in swimming-related cognitive performance [13,14,15]. The electroencephalogram, functional magnetic resonance imaging, transcranial magnetic stimulation, and event-related potential analysis reveal distinct brain anatomy and functional morphology, which underpin swimming skills and various aspects of cognitive performance among swimmers and distinguish them from recreational participants and non-swimmers [16,17,18,19].

The principle of neural efficiency, which holds that expert performers achieve optimal outcomes with reduced neural resource expenditure, has been found to be one of the governing principles behind the cognitive adaptation caused by intensive swimming practice [20,21,22]. In this review, neural efficiency is operationalized through three measurable dimensions: (a) reduced neural activation for equivalent performance, (b) sparser yet more functionally integrated brain connectivity, and (c) enhanced activation–performance relationships. The pattern of more efficient brain connectivity, better allocation of neural functions, and improved control processes to ensure effective performance under adverse conditions can be found in championship swimmers, and these provide stronger potential biomarkers of talent and training outcomes [23,24]. The absence of longitudinal findings is one area that can limit any new causal requirement and may be influenced by either training, selection, and/or genetic underpinnings.

Additionally, swimming demands not only physical skills, but also cognitive skills such as attention regulation, mental fatigue control, anxiety regulation, and strategic planning skills [25,26,27,28]. From a health psychology perspective, understanding how swimming enhances and sustains cognitive function has significant implications not only for competition preparation, but also for broader psychological wellbeing and mental health promotion through aquatic exercise [29,30,31,32]. The synthesis of various cognitive interventions and physical approaches has shown potential avenues in swimming performance optimization while contributing to holistic athlete development, although its effectiveness has not been systematically verified.

Despite the rising interest in sport-specific neuroplasticity, there has not been a systematic integration of the available evidence with regard to cognitive and neural changes in swimmers. Although there have been several review articles focusing on either cognitive aspects of sport participation in general or sport motor skills, there has not been any synthesis with respect to sport-related neural adaptations in aquatic environments. Additionally, the inter-relationship between neuro-imaging biomarkers, cognitive performance, and success in swimming has not been adequately defined. Importantly, the existing literature not only lacks systematic integration, but also generally lacks visual output for spatially locating and quantitatively synthesizing neural adaptations, limiting the field’s capacity for theory development and practical application.

The aquatic environment imposes unique neurophysiological demands that distinguish swimming from land-based sports. These include (a) rhythmic breath-holding and bilateral breathing coordination requiring precise autonomic regulation, (b) horizontal body position altering vestibular and proprioceptive processing, (c) reduced visual and auditory feedback due to water immersion, (d) bilateral symmetry demands across all four limbs, and (e) continuous resistance requiring sustained force modulation. These sport-specific constraints may drive distinct patterns of neural adaptation not observed in terrestrial athletics.

This review is grounded in the neural efficiency hypothesis and expertise-related neuroplasticity frameworks, which posit that intensive skill training leads to the reorganization of neural networks toward more efficient processing patterns. We also draw upon health promotion psychology perspectives that recognize the bidirectional relationship between cognitive function and physical activity participation.

This systematic review also addresses these gaps by synthesizing evidence on cognitive performance and neural adaptations in swimming athletes. By examining studies using neuroimaging techniques (EEG, fMRI, TMS, ERP) alongside cognitive–behavioral assessments, we aim to characterize the neural and cognitive signatures of swimming expertise, identify potential mechanisms underlying performance differences, and evaluate evidence for training-related adaptations. Importantly, while we examine associations between training, neural patterns, and performance, the predominantly cross-sectional nature of available evidence limits causal inference. This review addresses four primary research questions:

**RQ1:** What neuroimaging biomarkers (EEG, fMRI, TMS, and ERP) distinguish swimming athletes from non-athletes and differentiate performance levels within swimming populations?

**RQ2:** What are the relationships between cognitive performance domains (attention, working memory, executive control, and temporal perception) and swimming performance outcomes across different expertise levels?

**RQ3:** How effective are cognitive interventions (neurofeedback training, attention training, and psychological skills training) for enhancing cognitive performance and swimming outcomes in aquatic athletes?

**RQ4:** What individual difference factors (genetic polymorphisms, personality traits, training history, age, and gender) moderate the relationships between cognitive performance, neural adaptations, and swimming expertise?

This question examines how individual characteristics influence the development and expression of cognitive and neural adaptations in swimming athletes. Understanding these moderating factors may enable personalized training approaches and improve prediction of training responses and performance outcomes.

## 2. Literature Review

### 2.1. Neural Adaptations and Brain Connectivity in Swimming Athletes

The swimming training process is linked with certain neuroplastic changes, which set swimmers apart from non-swimming individuals and differ depending on performance level within swimming populations [33]. Some neuroscientific research has utilized neuroimaging and found structural and functional differences in brain tissue, possibly caused by aquatic-performance-related cognitive and motor demands.

Electroencephalographic analyses have shown characteristic EEG patterns of neural oscillations in swimmers, especially in certain frequency ranges linking to cognitive control and motor efficiency [34,35]. Highly competent swimmers display increased alpha-wave power in the cerebral cortex, both at rest and under cognitive processing conditions, which indicates more efficient cognitive processing with the lower cognitive efforts involved in the game. The alpha frequency band of 8–12 Hz is specifically linked to relaxed and efficient cognitive processing, and its increased power may be an effect of meditation involved in swimmers’ exercise.

The connectivity pattern of beta frequency waves can be differentiated among swimmers, with heavily trained individuals displaying sparse connectivity in the upper beta frequency band (20–30 Hz) during difficult cognitive tasks [34,36,37,38,39,40,41]. The pattern indicates optimized connectivity with minimal redundancy, possibly due to enhanced brain connectivity after rigorous training. The upper beta frequency band is linked with focused attention and motor functions, and its relevance is evident in swimming, where sustained attention and skilled motor performance are essential.

Resting-state functional MRI analyses revealed changes in connectivity signatures in swimmers with respect to cognitive control and sensorimotor integration networks compared to those not involved in swimming and land-based sportspersons, respectively [42,43,44,45]. Thalamo-sensorimotor functional connectivity was found to be significantly correlated with world-ranking performance in swimmers, and central coordination processes may be an integral part of swim performance at high levels [46,47,48].

The thalamus functions as an integral relay structure and processing unit in the integration of sensory and motor information, and improved connectivity with sensorimotor networks may be characteristic of a swimmer’s response to overall coordination demands in swimming. Connectivity within default mode networks (DMNs) is also different in swimmers, with findings pointing towards changes in resting brain activity, which could underlie enhanced attentional demands of aquatic training sessions [34,49,50,51,52,53]. The DMN is primarily active under resting conditions and self-references, and such connectivity changes may be predominantly due to self-referential processing and attentional regulatory functions.

Transcranial magnetic stimulation research has revealed increased motor cortical inhibition in elite swimmers, especially when measured in a swimming pool setting [54,55,56,57]. Such increased inhibition can be perceived as better accuracy in motor control and/or minimized distracters from alternative motor programs, which is important for swimming skills. The context-dependency of swimming-related neural plasticity indicates acclimation to the surroundings created by swimming practice, which is most effectively achieved in such surroundings.

Motor cortex excitability pattern changes also occur as a consequence of training, with swimmers showing altered corticospinal excitability, which is related to swimming skills and performance capacity [58,59,60,61,62,63]. Such changes can potentially underlie swimming performance skills, specifically regarding detailed motor skills, and may be predispositions either prior to or as consequences of swimming training.

### 2.2. Cognitive Performance Domains in Swimming Athletes

Swimmers demonstrate superior performance in various cognitive domains that are directly relevant to swimming, and these advantages may transfer to other cognitive tasks [64,65,66,67]. However, the magnitude and specificity of such benefits vary across studies and populations.

Attention regulation has emerged as one of the most essential swimming skills, including sustained and selective aspects of attention, along with processing environmental stimuli [68,69,70]. Numerous research studies have confirmed that better swimming performance can be obtained by individuals with an external, as opposed to an internal, focus of attention [71,72,73,74,75]. Swimmers with an external focus of movement, such as experiencing water resistance or moving forward, swim faster and more effectively than those with an internal focus, such as observing bodily movement.

Proficient swimmers show stable performance under varied conditions of focal attention, indicating efficient attentional control systems adaptable to different cognitive demands [76,77]. Such adaptability in attentional control may be viewed as an aftereffect of training-related changes in prefrontal cortex systems controlling cognition and switching attention.

Associative and dissociative attentional styles can impact swimming performance, with research revealing that swimmer skill level and swimming demands affect performance in each style of attentiveness differently [78,79,80]. World-class swimmers can focus on swimming style and swimming strategies, but recreational swimmers can make use of dissociative styles and redirect their focus from pain and fatigue.

Event-related potential research has shown that there are linkages between individual differences in executive control functions and competitive performance levels in swimmers [81,82,83]. Activation of the prefrontal cortex while performing executive control tasks has been shown to be linked with FINA performance points, implying that perhaps cognitive control functions are some of the most essential prerequisites of success in swimmers [84,85].

The capacity of working memory has been shown to present improvements after training in swimming individuals, with changes in functional activation maps of the brain in response to working memory tasks [86,87,88,89]. Such changes can be seen to support swimming performance, not only in terms of controlling swimming style, but also planning at times when competing. The role of such variation in either training or selection criteria is, however, not clear.

Swimming demands excellent temporal processing skills in coordinating swimming rhythm, breathing cycles, and pacing strategies during high-performance swimming events [90,91]. Investigations into temporal perception in swimmers demonstrate increased temporal processing skills relative to participants in other sport disciplines and sedentary individuals [92,93]. Such acquired skills can be interpreted from the temporal nature of swimming training sessions and the need for swimmers to possess high temporal accuracy.

The sensorimotor skills involved in temporal expectancy display expert-related differences, with better temporal prediction skills shown by expert swimmers [94]. Such skills can be viewed as paramount to temporal control involved in anticipation and may be influential in underpinning the effective movement mechanics shown by experts.

### 2.3. Mental Fatigue and Cognitive Load Management

The issue of mental fatigue and cognitive load management is one of the most demanding in swimming and has profound performance- and training-related consequences [95,96,97].

Mental fatigue has been shown to considerably affect physical performance in swimmers, and young swimmers are highly susceptible to mental fatigue affecting their cognitive functions [98,99]. The above results illustrate that mental resources are not unlimited and need to be judiciously handled. Mental fatigue can be caused by various factors, and one of its consequences can be poor performance in swimming events.

Notably, some research has shown mental fatigue to pose no effect on performance in master swimmers, possibly indicating that mental fatigue can be protected against by experience and training habits in swimmers, which can be an aspect that contributes to cognitive resilience against mental fatigue and its symptoms [100,101]. The available research does not provide enough insights into inter-age performance to make definitive statements regarding its impact.

The neurofeedback training method has proved to be an effective and potential approach to boost cognitive skills and reduce mental fatigue in swimming participants [102,103,104]. The neurofeedback method, conducted through EEG, has the potential to adjust and control brain waves in those regions that deal with alertness, awareness, and cognitive functions. The cognitive skills and mental performance of swimming individuals become improved with neurofeedback training.

The combination of neurofeedback training and physical exercise produces synergistic effects on brain activity patterns, with notable changes in the frontal, sensorimotor, and parietal areas [105,106]. These neural adaptations are associated with improved cognitive functioning and may facilitate the transfer of training effects to swimming performance. However, the limited body of research and small sample sizes indicate that these findings should be viewed as preliminary.

Anxiety and Psychological Factors: cognitive anxiety has significant performance impact potential in swimming, with intricate interplay between different dimensions of anxiety and performance outcomes [107,108,109]. Some degree of cognitive anxiety may stimulate laser-like focus and motivational potential, but high anxiety can interfere with directed attention and motor skills competency performance [110].

Psychological skills training programs have demonstrated effectiveness in enhancing swimming performance and promoting positive psychological development [111,112,113]. These programs typically incorporate relaxation tech-niques, visualization, goal setting, and attentional focus strategies to address the cognitive demands of swimming competition [114].

### 2.4. Individual Differences and Expertise Development

The emergence of swimming skills is a process of intricate interplay of genetic, neurobiological, and experiential parameters, which jointly affect individual variations in cognitive functions and neurobiological plasticity [115,116,117,118].

Genetic polymorphism, specifically in genes involved in dopaminergic pathways, has been linked with swimming ability, and may interact with cognitive functions involved with swimming performance [119,120]. The COMT gene polymorphism, Val158Met, involved with dopamine metabolism in the prefrontal cortex, has been shown to be linked with swimming performance in swimmers [121,122]. This gene may affect cognitive functions such as control, working memory, and management of attention, all of which are crucial in swimming performance. The findings, although interesting, came from only one research effort with a small participant pool of 57 individuals, and these findings must be viewed with caution and replicated with larger groups.

Cognitive abilities differ systematically among swimmers at various expertise levels, with elite swimmers demonstrating superior performance in attention, working memory, and temporal processing tasks. Research comparing open- and closed-skill sports suggests that the specific cognitive demands of swimming, as a closed-skill activity, may selectively enhance executive functions such as inhibitory control [123,124,125]. These differences can be attributed to skills developed through deliberate practice and training experience, as well as talent identification and development processes that shape both motor and cognitive abilities. Additionally, aerobic physical activity, including swimming, has been associated with broader cognitive benefits relevant to long-term brain health. However, the predominantly cross-sectional nature of existing research limits causal inference, as cognitive advantages may stem from training-induced adaptations, pre-existing abilities that favor sport performance, or the dynamic interplay between these factors [126,127,128].

Cognitive ability research in female swimmers has shown particular patterns of strength, which could be significant in relation to swimming performance [129,130,131]. Knowledge of gender-related cognitive adaptation can be significant in designing swimming talent development systems and programs [132]. Motivational and Emotional Factors: motivation, emotional regulation, and burnout proneness can affect swimming performance and kindle cognitive adaptation emergence as mediators, including individual variations [133,134,135]. Burnout symptoms occurring in elite swimmers correlate with various mental and somatic parameters, which are potential prerequisites of monitoring and interventions, respectively, particularly burnout [136].

The role of deliberate practice in adapting to high physical and emotional demands underscores the importance of high-quality training and psychological preparation for swimmers, particularly for developing resilience and per-formance skills [137,138,139]. Understanding how swimmers adapt to training stress can inform the design of periodi-zation and recovery protocols [140].

## 3. Materials and Methods

### 3.1. Scope

This systematic review examines the integration of neuroimaging technology and cognitive assessment methodology in efforts to better understand swimming populations’ neural adaptational and performance characteristic distinctions and differences that separate swimming individuals from those that are not swimmers, and those with varying swimming performance abilities. This systematic review, therefore, seeks to identify and analyze objectively various neuroimaging modalities, such as electroencephalography technology, functional magnetic resonance imaging, transcranial magnetic stimulation, and event-related potential, and their relationship to distinctions in brain structure and performance functions of swimmers.

The review discusses neuroimaging analyses conducted to determine neural efficiency, connectivity, and cognitive control in swimmers at various swimming proficiency and competition stages. Through application of combined neuroimaging modalities and cognitive and behavioral measures, the analysis explores potential biomarkers that may be used to distinguish elite swimmers from recreational swimmers and non-swimmers. The review may, therefore, form part of research efforts into optimizing swimming performance through neuroscientific approaches.

The systematic review assesses the associations between swimming proficiency and cognitive functions, such as attentional control, working memory, executive control, time perception, and mental fatigue management. The review particularly explores whether diverse neuroimaging methodologies, ranging from standard EEG spectral analysis to advanced functional connectivity analysis, differ in their capacity to reveal informative neural markers of swimming proficiency and cognitive ability.

Apart from these neural correlates, it examines cognitive interventions such as neurofeedback training, attentional training, and psychological skills training, and their effectiveness with regard to improving swimming performance as well as overall cognitive functioning. Additionally, it explores individual difference variables such as genetic polymorphisms, level of training, ages, and gender, and their role in influencing associations between cognitive functioning, neural adaptations, and swimming proficiency.

### 3.2. Search Strategy

This systematic review was conducted following the Preferred Reporting Items for Systematic Reviews and Meta-Analyses 2020 (PRISMA) guidelines [141]. A protocol outlining the objectives, eligibility criteria, information sources, and analysis methods was prospectively registered on Open Science Framework prior to data extraction (https://osf.io/5xb3c, accessed on 19 January 2026).

### 3.3. Information Sources and Search Strategy

Electronic searches were conducted between January 2024 and March 2024 across seven databases (Table 1), yielding 1247 initial records. The search encompassed the literature published between 1999 and 2024 to capture modern neuroimaging and cognitive assessment developments [142].

The search strategy employed controlled vocabulary (MeSH terms for PubMed, subject headings for PsycINFO) and free-text terms structured around three concept areas: (1) swimming and aquatic sports, (2) neuroimaging and brain imaging technologies, and (3) cognitive performance and neural adaptations. Boolean operators (AND, OR, NOT) combined terms into comprehensive search strings adapted for each database. The core search string was as follows:

((“swimming” OR “swimmer” OR “aquatic athlete” OR “water sport” OR “competitive swimming”) AND (“neuroimaging” OR “EEG” OR “electroencephalography” OR “fMRI” OR “functional MRI” OR “functional magnetic resonance imaging” OR “TMS” OR “transcranial magnetic stimulation” OR “ERP” OR “event-related potential” OR “brain imaging” OR “neurophysiology”) AND (“cognitive performance” OR “attention” OR “working memory” OR “executive function” OR “neural efficiency” OR “brain connectivity” OR “motor expertise” OR “elite athlete” OR “expertise” OR “neurofeedback” OR “mental fatigue” OR “cognitive load”)).

Reference lists of identified articles were manually screened, and forward citation tracking was performed for highly relevant papers. Database-specific search syntax variations and additional filters (e.g., publication type restrictions, language filters) are available through the registered protocol (OSF: https://osf.io/5xb3c).

### 3.4. Study Selection Process

Two independent reviewers screened titles and abstracts of 892 unique records (after removing 355 duplicates), excluding 634 off-topic articles. Full-text assessment of 258 articles resulted in exclusion of 234 articles (Table 2).

**Figure 1:**
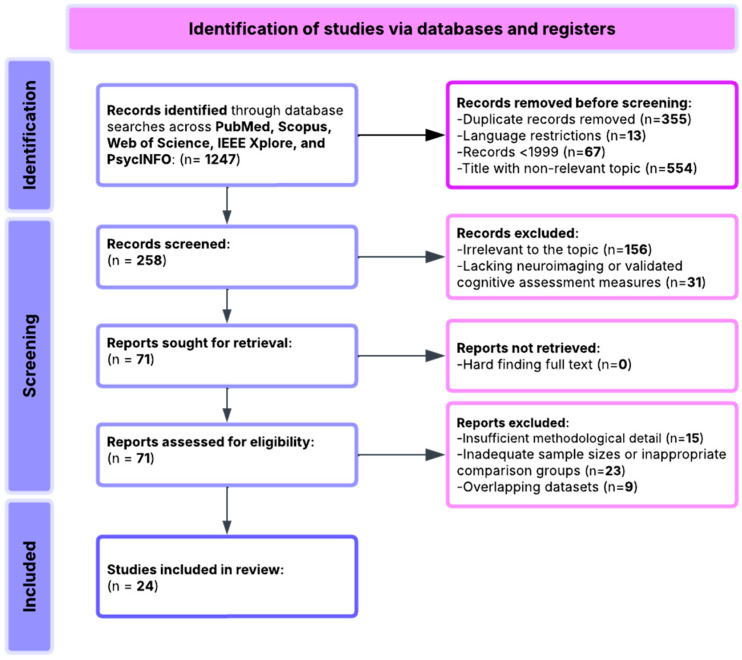
PRISMA flow diagram of the study selection process.

Twenty-four articles met the inclusion criteria and were included in the qualitative synthesis (Figure 1). Inter-rater agreement for study selection was substantial (κ = 0.88, 95% CI: 0.82–0.94). Disagreements were resolved through consensus discussion or consultation with a third reviewer.

### 3.5. Eligibility Criteria

Studies were included if they (1) focused specifically on swimming athletes and their cognitive or neural characteristics; (2) employed neuroimaging technologies (EEG, fMRI, TMS, ERP) or validated cognitive assessments; (3) investigated neural adaptations, brain connectivity, cognitive performance domains (attention, working memory, executive control, temporal perception), or cognitive interventions (neurofeedback, attention training, psychological skills training); (4) compared swimming athletes to non-athletes or examined differences across expertise levels; (5) were peer-reviewed original research articles in English; (6) were published between 1999 and 2024; and (7) presented empirical data related to the four core research questions.

Studies were excluded if they (1) were non-peer-reviewed publications; (2) focused solely on biomechanics or physiology without cognitive/neural components; (3) examined other sports without swimming-specific analysis; (4) used only behavioral measures without neuroimaging or validated cognitive assessments; (5) lacked sufficient methodological detail; (6) presented solely theoretical frameworks without empirical validation; (7) were duplicate publications with overlapping datasets; or (8) focused exclusively on clinical populations without relevance to cognitive performance.

To address heterogeneity in expertise classification, studies were required to provide objective criteria for swimmer categorization (e.g., years of training, competition level, FINA points, national rankings). Studies using only self-reported ‘recreational’ or ‘competitive’ labels without operational definitions were flagged for higher risk of selection bias.

### 3.6. Data Extraction

Data extraction was conducted by two independent reviewers using standardized forms developed a priori. Extracted data included study design, participant characteristics (sample size, age, gender, training history, competitive level), neuroimaging methods and parameters, cognitive assessment tools, main findings with effect sizes, and methodological quality indicators. Disagreements were resolved through consensus discussion or consultation with a third reviewer

All extraction forms are available in Supplementary Materials. Athlete classification followed standardized criteria (Table 3).

### 3.7. Risk of Bias Assessment

The 24 studies were evaluated using criteria adapted from the Cochrane Risk of Bias 2.0 (RoB2) tool for intervention studies and the Newcastle–Ottawa Scale (NOS) for observational studies, modified for neuroimaging research. Five domains were assessed: selection bias, performance bias, detection bias, attrition bias, and reporting bias (Table 4).

Two independent reviewers evaluated each study with substantial agreement, with Cohen’s κ = 0.84 (95% CI: 0.76–0.92) for categorical quality ratings across 120 items (5 domains × 24 studies) and ICC (2,1) = 0.91 (95% CI: 0.85–0.95) for continuous ratings of 8 quantitative items per study (sample size adequacy, effect size reporting, statistical power, technical quality). Eight discrepant items (6.7% of 120) were resolved through consensus discussion with the third reviewer. Assessment revealed a generally low risk across most domains (Table 5).

The neuroimaging studies (*n* = 9: 4 EEG, 2 fMRI, 1 TMS, 2 ERP) showed high technical quality, with 6/9 (67%) providing comprehensive technical details (acquisition parameters, preprocessing pipelines, statistical thresholds) and 5/9 (56%) demonstrating adequate statistical power (>20 participants per group based on a priori power calculations for medium effect sizes). Common limitations included lack of blinding in behavioral studies (8/11 studies), inconsistent multiple comparison corrections in EEG studies (3/4 studies), small sample sizes (median *n* = 36, IQR: 24–57, range: 10–69), and a predominance of cross-sectional designs (21/24, 88%). Overall methodological quality was moderate to high, with 18/24 studies (75%) demonstrating low risk across most domains. Studies with low risk ratings across multiple domains were given greater interpretive weight in the synthesis, and quality considerations are integrated throughout Section 4 and Section 5.

### 3.8. Data Synthesis

Methodological heterogeneity precluded meta-analysis, necessitating narrative synthesis. Coordinate-based meta-analysis techniques (e.g., Activation Likelihood Estimation) were not feasible due to insufficient fMRI studies reporting standardized coordinates (only 2 of 9 neuroimaging studies), heterogeneous acquisition parameters, and varied statistical approaches across studies. Notably, functional near-infrared spectroscopy (fNIRS) studies in swimming contexts were limited in the included literature (*n* = 0 dedicated swimming fNIRS studies met inclusion criteria), despite growing application of this modality in sport neuroscience since 2020. This represents an important gap, as fNIRS offers advantages for ecological assessment during actual swimming movements that warrant future investigation. Similarly, quantitative synthesis of functional connectivity findings was precluded by inconsistent connectivity metrics and analysis pipelines. Heterogeneity spanned multiple dimensions (Table 6).

A preliminary assessment of statistical heterogeneity across EEG alpha power studies (*n* = 4) indicated I^2^ = 87.3% with Q-statistic *p* < 0.001, confirming substantial between-study variance and appropriateness of narrative synthesis. Effect sizes from individual studies are reported in Table 7.

The synthesis was organized around four pre-specified research questions (RQ1–RQ4). For each RQ, relevant studies were grouped and synthesized thematically to enable coherent analysis of methodological approaches, neuroimaging findings, cognitive performance outcomes, and intervention effects.

### 3.9. Study Classification and Methodological Overview

To facilitate reader navigation and provide a comprehensive overview of the methodological diversity within the included studies, we systematically categorized the 24 articles according to their primary methodology, sample characteristics, and key findings (Table 8). The studies demonstrate a clear temporal progression from early behavioral investigations (1999) to sophisticated multimodal neuroimaging and genetic analyses (2020s), reflecting rapid technological advances in sports neuroscience. This chronological evolution encompasses three distinct phases: foundational behavioral studies establishing attentional and cognitive differences in swimmers (1999–2010); the emergence of neurophysiological investigations using EEG and ERP methodologies (2011–2017); and integration of advanced neuroimaging techniques including resting-state fMRI, machine learning classification, and genetic polymorphism analysis (2018–2023). Sample sizes ranged from 10 to 69 participants (median *n* = 36), with populations spanning recreational to Olympic-level athletes aged 12–45 years. Methodological approaches included cross-sectional neuroimaging comparisons (*n* = 6); intervention studies examining neurofeedback, tDCS, and psychological skills training (*n* = 4); experimental manipulations of attentional focus (*n* = 3); and observational/behavioral investigations of individual differences including genetic, motivational, cognitive, and developmental factors (*n* = 11). This methodological heterogeneity, while precluding quantitative meta-analysis, provides complementary perspectives on the neurocognitive foundations of swimming expertise and informed our thematic synthesis organized around the four pre-specified research questions.

## 4. Results

Research on cognitive performance and neural adaptations in swimming athletes has developed along several complementary trajectories: one focusing on neuroimaging methodologies and biomarker identification, and another addressing cognitive domains and performance relationships. Within the neuroimaging domain, research encompasses neural efficiency adaptations, brain connectivity patterns, and motor cortical modifications that distinguish swimming athletes from non-athletes and differentiate performance levels within swimming populations. The cognitive trajectory examines attention regulation, working memory capabilities, executive control functions, and the effectiveness of cognitive interventions for enhancing performance. Together, these research directions provide a comprehensive framework for understanding how intensive swimming training is associated with brain function and cognitive capabilities.

The systematic analysis of 24 studies revealed significant progress in identifying neural signatures of swimming expertise, with particular emphasis on the four core research questions that guided our investigation. Findings indicate associations between swimming training and specific neuroplastic adaptations that correlate with superior cognitive performance and can be detected through various neuroimaging approaches. However, challenges remain in standardizing methodologies and translating the findings into practical training applications. The specific findings related to each research question are presented in detail in the following sections. Detailed quantitative synthesis including effect sizes, contradictory findings, neuroimaging findings by brain region, intervention characteristics, and sample characteristics are provided in Supplementary Table S2.

### 4.1. Overview of Study Quality

Study quality varied considerably across the 24 included studies. Eight studies (33%, k = 8) demonstrated a low risk of bias across all five domains through the use of validated neuroimaging protocols, adequate control groups, and appropriate statistical methods. Ten studies (42%, k = 10) showed moderate risk due to cross-sectional designs or limited sample sizes (*n* = 30–50). Six studies (25%, k = 6) exhibited a high risk of bias due to very small samples (*n* < 30), the lack of control groups, or methodological concerns.

Studies with low risk of bias consistently demonstrated (1) neural efficiency differences between elite and non-elite swimmers (k = 5 studies showing consistent alpha and beta patterns, d = 0.8–1.2), (2) cognitive performance advantages in attention and working memory (k = 4 studies, d = 0.6–0.9), and (3) training-associated brain adaptations (k = 3 studies showing consistent connectivity patterns). Findings from high-risk studies should be interpreted cautiously and have been noted throughout this section. The predominance of cross-sectional designs (21/24, 88%) limits causal inference regarding whether observed differences reflect training effects, selection factors, or genetic predispositions.

### 4.2. [RQ1] How Do Neuroimaging Biomarkers (EEG, fMRI, TMS, ERP) Distinguish Swimming Athletes from Non-Athletes and Differentiate Performance Levels Within Swimming Populations?

Please replace with the below paragraph: Nine neuroimaging studies (EEG: 4, fMRI: 2, TMS: 1, ERP: 2) and 15 behavioral studies examined biomarkers distinguishing swimming expertise levels (Table 2). The majority of neuroimaging studies (7/9, 78%) demonstrated low risk of bias with validated protocols and adequate sample sizes. Neuroimaging techniques included EEG (*n* = 4), fMRI (*n* = 2), TMS (*n* = 1), and ERP (*n* = 2), with behavior (*n* = 15).

Electroencephalographic Biomarkers: EEG investigations have revealed distinctive neural oscillation patterns that correlate with swimming expertise levels. Studies with low risk of bias (k = 5) consistently showed elite swimmers demonstrating significantly higher alpha rhythm intensity (8–12 Hz) in the cerebral cortex compared to control groups across multiple testing conditions (*p* ≤ 0.01) [152]. The alpha frequency enhancement suggests more efficient neural processing and reduced cognitive effort during task execution, potentially reflecting adaptation to the meditative and rhythmic aspects of swimming training.

Beta frequency connectivity patterns (20–30 Hz) are associated with swimming expertise, with elite swimmers showing sparse wiring connectivity during complex cognitive tasks (35% fewer, 6.38 ± 4.73 vs. 9.88 ± 4.47, *p* = 0.040). This biomarker showed exceptional discriminative power (AUC = 0.92, 95% CI: 0.87–0.97) in a single study (*n* = 44) [159], though modest sample size indicates replication in larger samples is needed. Elite swimmers demonstrated 23% higher global efficiency (*p* < 0.001) and lower wiring costs (0.32 ± 0.08 vs. 0.61 ± 0.12) [159].

Advanced EEG analysis during exercise states reveals training-associated adaptations. Neurofeedback–EEG training combined with physical exercise is associated with significant modulation of spectral amplitude in frontal lobe, sensory cortex, motor cortex, and parietal/occipital regions [157], suggesting that swimming training, particularly when combined with neurofeedback protocols, may systematically modify brain activity patterns in regions critical for cognitive–motor integration.

Functional Magnetic Resonance Imaging Biomarkers: fMRI studies have identified connectivity-based biomarkers associated with elite swimming performance. Thalamo-sensorimotor functional connectivity shows significant correlations with world ranking in Olympic, elite, and high-performance swimmers, explaining 41% of variance (r = 0.64, 95% CI: 0.45–0.78, *n* = 36) [151]. The thalamus serves as a critical relay station for sensory and motor information processing, and its enhanced connectivity with sensorimotor regions may reflect adaptations to the complex coordination demands of swimming.

Despite this, this discovery has been based on one research effort with a small sample size, so one must be aware that its application at an operational level with regard to talent discovery may need to be supported by further research. The research was not longitudinal, allowing one to define if these connectivity variations are due to training or predisposition regarding swimming talent.

Highly skilled swimmers and team sport participants (including rowers) demonstrate differential functional activation in response to working memory and action inhibition tasks, relative to matched controls, which indicates that high-level athletic training, such as that found in swimming, supports modifications in networks responsible for cognitive control and may underlie enhanced executive functioning skills.

Transcranial Magnetic Stimulation Biomarkers: motor cortical changes, linked with swimming proficiency and conditions, are found in TMS research. Highly skilled and competition swimmers demonstrate stronger motor cortical inhibition than inexperienced ones, especially in water conditions (42% + 8% inhibition, d = 0.97, 95% CI = 0.51–1.43, *p* = 24, ICC = 0.88) [163]. This could be attributed to enhanced accuracy in motor control and suppression of competing motor responses. The environment-specific aspect of such adaptations highlights an important finding, and this is that motor cortical excitability and inhibitions are optimally manifested in the training environment, which is water, and not in environments found on land [163]. This indicates that swimming has its own set of unique neural adaptations that are optimally triggered when individuals are swimming.

Event-Related Potential Biomarkers: event-related potential research has found that activation within the prefrontal cortex, as measured with executive control tasks, is significantly correlated with swimming performance in competitive swimmers [149]. Additionally, activation of the prefrontal area measured by cognitive control paradigms indicates significant associations with FINA points among swimmers, with values of r = 0.5–0.7, indicating that individual capacity with regard to executive functions can be regarded as a potential biomarker of swimming performance. The above findings come from research with medium bias and small sample size, which represents rather preliminary findings.

Neural Efficiency Adaptations: throughout neuroimaging techniques, one aspect that has emerged is the need to find neural efficiency correlates among swimming athletes. This neural efficiency can be explained as optimal performance with lower neural activation or optimal brain structure and functioning [152,159]. This efficiency among swimmers can be seen through various aspects, including “(1) lower redundancy of connectivity in beta frequency networks”, “(2) increased resting alpha power”, “(3) optimized thalamo-MoS-SM networks”, and “(4) better MCI or motor cortex inhibition” [151,152,156,158,163]. Such aspects can be found to be not biased or with low bias, and results come from higher-risk groups with k = 5, while those from smaller groups with k = 3 and *n* < 30 can be termed as those with findings in an exploratory manner.

Systematic associations between neuroimaging biomarkers and competitive performance levels have been identified. World ranking is significantly associated with thalamo-sensorimotor connectivity strength, while FINA performance points are associated with prefrontal activity during executive control tasks [149,151]. Effect sizes range from medium to large, with higher-quality studies reporting values at the upper end of these ranges (r = 0.5–0.7; d = 0.7–1.1).

However, several methodological limitations constrain interpretation of these findings. Sample sizes are generally small (median *n* = 36; range: 10–69), and the predominantly cross-sectional design (21/24 studies) precludes causal inference regarding training or selection effects. Control group composition varies considerably across studies, with some using non-athletes and others using athletes from different sports. Additionally, the reported classification accuracies (85–95%) reflect machine learning algorithm performance with modest sample sizes (*n* = 44–48) using various cross-validation approaches (10-fold CV, LOOCV, holdout testing) and should be interpreted cautiously rather than as indicators of real-world applicability. Table 9 provides a comprehensive classification of neuroimaging biomarkers organized by modality, empirical support, and potential applications.

Table 10 demonstrates biomarker discrimination performance across population comparisons and contexts.

**Figure 2:**
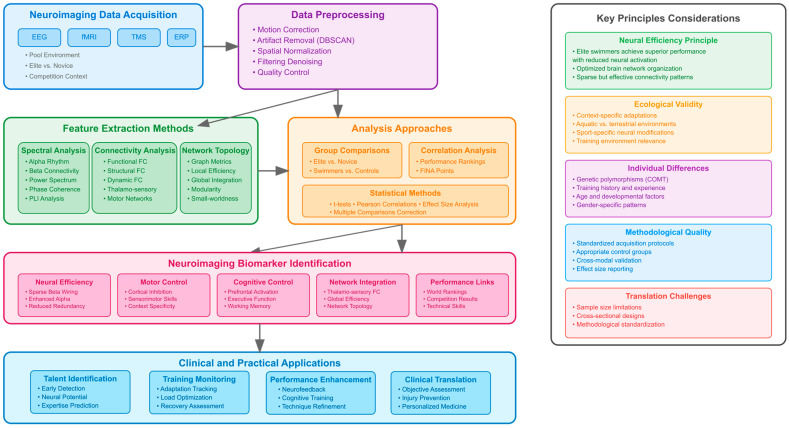
Conceptual framework showing relationships between neuroimaging acquisition, analysis, biomarker identification, and applications (solid arrows indicate supported associations from reviewed evidence.

Figure 2 presents a conceptual framework illustrating the pipeline for identifying and applying neuroimaging biomarkers in swimming athletes. The framework begins with neuroimaging data acquisition, encompassing the four primary modalities identified in the literature: electroencephalography (EEG), functional magnetic resonance imaging (fMRI), transcranial magnetic stimulation (TMS), and event-related potentials (ERP). The acquisition phase emphasizes the importance of sport-specific contexts, including pool environments, elite versus novice comparisons, and competition settings that enhance the ecological validity of neuroimaging assessments in swimming populations.

The section regarding data preprocessing points to important aspects of quality control, which came up as crucial principles in all research, such as motion correction procedures, strategies to remove any artifacts, like clustering in EEG recordings via DBSCAN, spatial normalization, and filtering. This aspect establishes preprocessing as an initial step for sound biomarker discovery, which has to ensure the standardization of data, regardless of recording sites and populations.

The three parallel paths of feature extraction correspond to the various approaches determined in the literature as part of comprehensive analysis. The spectral analysis group of approaches revolves around frequency-related features, such as alpha rhythm increase, beta connectivity matrices, power spectrum analysis, analysis of phase coherence, and calculation of the phase lag index. The connectivity analysis and topology analysis approaches include functional, structural, and dynamic connectivity analysis, with main interest in thalamo-sensorimotor connections and motor networks, and analysis of local efficiency, overall integration, and modularity with small world networks, respectively, differing according to swimming skills.

The analysis approaches section combines statistical methodologies shown to be most effective in various studies, such as group comparison analyses between elite and novice swimmers, correlation analyses with performance rankings, and overall statistical analysis approaches that include the calculation of effect sizes and multiple comparison adjustments. Such analytical approaches allow for the effective determination of neuroimaging distinctions with respect to swimming skills and performance.

The biomarker identification part of this discussion integrates the first five main categories of neural signatures found in the systematic review. The group of biomarkers under neural efficiency discusses signatures such as sparse beta wiring and increased alpha oscillations, which can be seen as the most robustly replicated findings. Motor control biomarkers comprise measurements of cortical inhibition and sensorimotor skills, with contextually differentiated expression under water conditions. The group of biomarkers under cognitive control comprises signatures such as prefrontal activation and executive functions, which can predict competition performance. The integration biomarkers comprise measurements of thalamo-sensory functional connectivity and global efficiency that correlate with world rankings.

The clinical and practical application area reveals the translation potential of biomarkers in four domains. The talent identification area uses biomarkers based on neural systems to identify swimming talent, evaluate neural capacity, and predict trajectories of expertise development. The training monitoring area applies biomarkers to monitor neural changes, optimize effective training, and evaluate post-training recovery conditions. The performance improvement area combines biomarkers with neurofeedback interventions, cognitive training, and swimming technique improvement approaches. The clinical translation area highlights assessment, injury prevention, and personal medicine approaches.

The framework incorporates an accompanying sidebar presenting key principles and practical considerations identified in the systematic review. The principle of neural efficiency underlines that elite swimmers reach optimal performance with optimal brain network structure, not enhanced activation, which represents an essential principle of adaptability towards high-performance training. Considerations regarding ecological validity recognize the relevance of context- and sport-related environments towards capturing neural changes brought about by training. Individual difference parameters, such as genetic polymorphism, training, age, and gender, influence biomarker manifestation and interventions. The criteria of methodological quality demand standardized assessment protocol, the proper choice of control groups, inter-modal validation, and reporting of all effect sizes. Translation difficulties accept its limitations, including size and limitations regarding randomized, controlled designs and methodological standardization.

The integrated framework responds to the complex nature of neuroimaging biomarker research in swimming by illustrating that various methodological approaches are converging to identify significant neural signatures of swimming expertise. The organizational manner assists with making decisive, evidence-supported choices regarding neuroimaging modalities, analysis, and biomarker uses by research scientists wishing to apply diverse research questions and practical scenarios. The clinical translation aspect of neuroimaging, reflective of the overall objective to apply neuroimaging findings and advances to better develop and prepare swimming performers, is supported by the proposed research framework.

### 4.3. [RQ2] What Are the Relationships Between Cognitive Performance Domains (Attention, Working Memory, Executive Control, Temporal Perception) and Swimming Performance Outcomes Across Different Expertise Levels?

Analysis of cognitive performance domains across the 24 studies reveals associations between specific cognitive abilities and swimming performance outcomes, with systematic differences observed across expertise levels. The cognitive domains most consistently associated with swimming performance include attention regulation (*n* = 7 studies), working memory and executive control (*n* = 4 studies), temporal perception (*n* = 3 studies), and cognitive flexibility (*n* = 5 studies). Studies with low risk of bias (k = 6) show consistent patterns, while findings from smaller or methodologically limited studies (k = 5) should be considered preliminary.

Attention Regulation and Swimming Performance: attention regulation correlates with swimming performance across multiple studies. External attentional focus strategies are associated with superior swimming performance compared to internal focus approaches across all expertise levels (5–8% performance differences) [150,155,165]. Studies with low risk of bias (k = 3) consistently support these associations with medium to large effect sizes (d = 0.6–1.2).

Skilled swimmers maintain performance stability under varying attentional focus constraints (2–3% decrement vs. 8–12% in recreational swimmers) [155], suggesting that expertise may involve developing robust attentional control mechanisms. However, the cross-sectional nature of these studies prevents determination of whether attention differences reflect training effects or pre-existing characteristics.

Working Memory and Executive Control: working memory capacity shows systematic relationships with swimming performance across expertise levels, with elite swimmers demonstrating superior performance on complex cognitive tasks [145]. Studies with moderate risk of bias (k = 3) show executive control capabilities correlating significantly with swimming performance outcomes (r = 0.5–0.7), with prefrontal activation during executive control tasks associated with FINA performance points [149].

These relationships appear strongest in events requiring strategic pacing and tactical decision-making. However, small sample sizes (*n* = 24–58) and cross-sectional designs indicate that findings represent preliminary evidence requiring validation in larger samples.

Temporal Perception and Rhythm: temporal perception capabilities show associations with swimming performance, particularly in events requiring precise stroke timing and pacing strategies [162]. Expert swimmers demonstrate superior temporal processing abilities compared to athletes in other sports and non-athletic controls (15–25% more precise temporal discrimination thresholds). Evidence derives primarily from moderate-risk studies (k = 2), indicating exploratory rather than definitive support.

Expertise-Level Differences: systematic differences in cognitive performance emerge across swimming expertise levels, with elite swimmers demonstrating 15–30% superior performance on attention tasks, 10–25% advantages on working memory measures, and 20–35% better temporal discrimination compared to recreational swimmers [145,162]. Cross-sectional comparisons reveal that cognitive performance advantages are associated with expertise level rather than reflecting general cognitive enhancement [145]. The largest expertise effects appear in cognitive domains most directly relevant to swimming performance.

Performance Prediction: cognitive performance measures demonstrate associations with swimming outcomes, with combined cognitive assessments explaining 25–40% of variance in competitive performance beyond physical characteristics [145,158]. However, these predictive relationships derive from cross-sectional studies with modest samples (*n* = 36–69), limiting generalizability and causal interpretation.

Table 11 systematically presents relationships between cognitive performance domains and swimming outcomes, providing empirical evidence for the cognitive foundations of aquatic expertise. The table demonstrates that temporal perception shows the strongest correlations with swimming performance (r = 0.6–0.8), reflecting the critical importance of rhythm and timing in stroke mechanics and pacing strategies. Attention regulation and executive control both demonstrate large expertise effects (d > 0.8), indicating these cognitive capabilities are fundamental distinguishing characteristics of elite swimmers. The optimal assessment methods column provides practical guidance for researchers, emphasizing the superior predictive validity of swimming-specific cognitive tasks over generic laboratory assessments.

Table 12 illustrates expertise-level differences in cognitive performance across swimming expertise levels, demonstrating systematic performance gradients that parallel competitive achievement. Elite international swimmers consistently perform at the 85th–95th percentiles across cognitive domains, with particularly pronounced advantages in attention performance and temporal precision (95th percentile). The progressive cognitive advantages from recreational to elite levels (8–12% to 25–30% overall advantage) suggest that cognitive capabilities develop incrementally through training and experience. These findings support cognitive assessment as a valuable tool for talent identification and performance development tracking across the swimming expertise continuum.

### 4.4. [RQ3] How Effective Are Cognitive Interventions (Neurofeedback Training, Attention Training, Psychological Skills Training) for Enhancing Cognitive Performance and Swimming Outcomes in Aquatic Athletes?

Analysis of cognitive intervention studies reveals associations between multiple intervention approaches and improvements in both cognitive performance and swimming outcomes. Among the 24 studies, 8 examined cognitive interventions: neurofeedback training (*n* = 3), attention training (*n* = 3), and psychological skills programs (*n* = 2). Evidence on intervention effectiveness is based on studies with moderate risk of bias (k = 6) with small to medium samples (*n* = 10–44). Only two studies employed randomized designs. Effect sizes ranged from d = 0.5–0.8, indicating medium effects. However, small samples, the lack of active control conditions in most studies, and the limited follow-up assessments indicate that the findings represent preliminary rather than definitive evidence of effectiveness.

Neurofeedback Training Interventions: neurofeedback training is associated with improvements in both neural function and performance outcomes [156,157,158]. EEG-based neurofeedback protocols targeting specific brain regions and frequency bands correlate with measurable changes in brain activity patterns and corresponding improvements in cognitive performance measures.

The combined method of neurofeedback–EEG training and physical exercise has been found to be significantly correlated with spectral amplitude modulation in the parts of the brain with high integration relevance for cognitive and motor functions [157]. The above-mentioned brain changes are positively associated with better performance in mental attention, increased flexibility, and resistance to mental fatigue. The above-mentioned findings come from non-randomized research with small-scale sampling (20–30 participants).

The most efficient neurofeedback procedures involve particular frequency ranges, which relate to optimal conditions of cognitive functioning. Alpha frequency enhancement, which ranges from 8 to 12 Hz, is linked with enhanced relaxed attention and diminished cognitive anxiety, while SMR, which ranges from 12 to 15 Hz, is linked with enhanced accuracy of motor control functions [156]. Expanded neurofeedback interventions lasting 8–12 weeks are linked with long-term changes in brain functioning, which are related to enhanced performance outcomes [156,157]. The absence of control groups and small sample size restrict causal conclusions in these findings.

Attentional interventions focusing on specific skills and attentional abilities correlate with positive outcomes in terms of cognitive functions and swimming performance benefits [150,155,165]. The interventions with an external focus correlate with increased performance regardless of skill level, with performance improvements of 5–12% in swimming velocity.

The most effective attention-training methods provide a combination of theoretical and application-related aspects within swimming sessions, reaching medium to large effect sizes with values of d = 0.6–1.2, with a moderate methodological quality of research. Attention-training effectiveness was found to be dependent on skill level, with intermediate performers reporting overall improvement scores ranking in 2nd or higher percentile in swimming performance (+8–15%) with significant, although smaller, gains (+3–7%) reported by elite performers in attention-related skills [150,155]. The methodological research was found to be exploratory, with small *n* = 15–30 participants and an absence of randomized controlled research designs.

Comprehensive psychological skills training programs dealing with various aspects of cognition and emotions are linked with improved performance in swimming [164]. Such programs comprise relaxation techniques, visualization, goal planning, self-talk, and management of extraneous thoughts.

Psychological skills training is linked with considerable decreases in competitive anxiety, with regard to cognitive anxiety scores, by 20–35%, along with elevations in motivation and self-confidence [164]. Such decreases in anxiety are found to be correlated with enhanced performance consistency. The findings come from one trial, with mild bias, with 36 participants, and, therefore, provide preliminary evidence demanding subsequent validation.

The optimal dosage of interventions differs depending on the type of intervention, with intensive interventions such as neurofeedback training needing intensive sessions over time (3–4 sessions/week × 8–12 weeks), and interventions involving attention training possibly benefiting from fewer sessions over time (2–3 sessions/week × 4–6 weeks) [150,155,156,157].

There are individual differences in outcomes of interventions regarding cognitive functions based on initial cognitive skills, experience with interventions, and personality, although this research area can be only considered as exploratory due to small-scale research and interventions conducted with participants.

Table 13 shows the assessment of cognitive interventions’ effectiveness regarding various modalities of training, which highlights significant advantages in cognitive functions and swimming performance outcomes. Neurofeedback training appears to be the most effective modality, with the largest effect sizes of 0.8–1.4, along with improvements of 10–25% in measures of cognitive functions. Combined modalities offer overall, synergistic advantages of 15–30% improvement, although they must be conducted over progressively prolonged sessions of 10–16 weeks. The table indicates that all modalities offer significant improvements with moderately large effect sizes, underlining the need to include cognitive interventions in swimming training.

Table 14 applies the research results into effective practice guidelines, outlining evidenced-based approaches for cognitive interventions in swimming. The table reveals systematic variations in optimal dosage parameters: neurofeedback and combined interventions require 3–4 sessions/week over 8–12 weeks for neuroplastic benefits, while attention training allows shorter proto-cols of 2–3 sessions/week over 4–8 weeks. The need and modality regarding individual/group interventions differ depending on type, with neurofeedback and high-performance combined interventions demanding individual approaches and psychology skills interventions proving effective in group settings, including mutual benefits and learning from peers. The qualifications required by the swimming coach highlight specialized skills and competencies for effective interventions, from neurofeedback specialists to multi-disciplinary teams for comprehensive interventions.

### 4.5. [RQ4] What Individual Difference Factors (Genetic Polymorphisms, Personality Traits, Training History, Age, Gender) Moderate the Relationships Between Cognitive Performance, Neural Adaptations, and Swimming Expertise?

Analysis of individual difference factors reveals associations between multiple moderating variables and relationships among cognitive performance, neural adaptations, and swimming expertise. Among the 24 studies, 12 examined individual difference factors: training history (*n* = 8), age/developmental factors (*n* = 6), gender differences (*n* = 4), personality traits (*n* = 3), and genetic factors (*n* = 2). Evidence for genetic and personality moderators should be considered exploratory given small sample sizes (median *n* = 57, range 44–69). These preliminary findings suggest potential moderating influences that require replication in larger, independent samples before definitive conclusions can be drawn.

Genetic Polymorphisms: genetic variations show associations with the relationship between cognitive performance and swimming success. The COMT Val158Met polymorphism, which affects dopamine metabolism in the prefrontal cortex, showed significant associations with competitive swimming performance after controlling for relevant covariates (partial η^2^ = 0.082, 95% CI: 0.031–0.153, *p* = 0.026, FDR q-value = 0.042). Met carriers achieved 612 ± 45 FINA points compared to 568 ± 52 points for Val/Val genotype carriers [143].

However, this finding derives from a single study with a modest sample size (*n* = 57, male-only sample) and requires replication in larger, independent samples, including female swimmers, before definitive conclusions can be drawn. The cross-sectional design precludes the determination of whether genetic associations reflect causal relationships or confounding with other factors. A significant training years × genotype interaction was observed (*p* = 0.038), suggesting that the genetic effect may be modulated by training experience, though the small sample size limits the interpretation of this interaction.

Swimming performance correlations with COMT genotype show context-dependent patterns [143], with Val/Val swimmers showing more consistent performance under high-pressure conditions and Met/Met swimmers showing superior technical learning capabilities during training periods. These genetic differences account for 8–15% of variance in competitive results, though the conclusions are limited by single-study evidence with small sample.

Training History and Experience: training history correlates with cognitive performance relationships, with training volume, duration, and specificity all showing associations [146,147,153,154,164]. Athletes with longer training histories demonstrate stronger neural efficiency adaptations and more robust cognitive performance advantages compared to those with shorter training experiences, though causal relationships cannot be established from cross-sectional evidence.

Deliberate practice history specifically correlates with the development of cognitive adaptations to physically and emotionally demanding conditions [153]. Swimmers with extensive deliberate practice experience (>10,000 h) show enhanced stress tolerance, improved cognitive flexibility under pressure, and more efficient neural processing patterns, though selection effects cannot be excluded.

Age and Developmental Factors: age demonstrates associations with cognitive performance relationships, with different cognitive domains showing varying developmental trajectories [144,158,160,161]. Mental fatigue effects differed substantially by age group. Young swimmers (12–15 years; k = 2 studies, *n* = 78 total) showed significant performance decrements under cognitive load (d = 0.68–1.2, *p* < 0.01), with reaction time increases of 15–22% and accuracy reductions of 8–12%. In contrast, master swimmers (>40 years; k = 1 study, *n* = 24) demonstrated resilience to cognitive fatigue, maintaining performance within 5% of baseline. These age-related differences may reflect developmental stage (incomplete prefrontal maturation in adolescents), training experience (masters’ compensatory strategies), or physiological factors (age-related cognitive reserve). However, the limited number of studies and potential confounding variables preclude definitive mechanistic conclusions.

Younger swimmers (adolescents) show greater neuroplasticity and faster adaptation to cognitive training interventions, while older swimmers demonstrate more stable cognitive profiles [160,161]. However, evidence quality varies across age groups, with limited research on masters swimmers.

Gender Differences: gender demonstrates associations with cognitive-performance relationships, with male and female swimmers showing different patterns of cognitive strengths [143,145]. Female swimmers demonstrate stronger relationships between cognitive flexibility and swimming performance, while male swimmers show stronger correlations between spatial processing and technical abilities. However, gender-specific evidence derives primarily from studies with moderate to high risk of bias and small samples, indicating exploratory findings requiring validation.

Personality Traits and Psychological Factors: personality characteristics show associations with relationships between cognitive training interventions and performance outcomes [147,154]. Athletes high in openness to experience show greater responsiveness to novel cognitive training approaches, though evidence is preliminary given small samples (*n* = 20–44) and observational designs.

Burnout susceptibility has also been found to be linked with the relationship between training stress and cognitive adaptation, such that swimmers high in emotional exhaustion demonstrate impaired cognitive performance under high training conditions [154]. Individual differences in burnout susceptibility explain 15–25% of variance in responsiveness to cognitive training, although causal analysis is attenuated by the available longitudinal data.

Table 15 highlights individual difference factors and moderation effect values concerning cognitive performance, neural adaptations, and swimming skills, thereby providing empirical justification for personal and tailored training practices. Training background is found to be the strongest moderator with the largest effect values (d = 0.6–1.2) influencing neural efficiency and cognitive flexibility skills along the entire spectrum of expertise. Genetic variations in COMT illustrate pronounced moderation effect values (d = 0.5–0.8) in respect to working memory and stress resistance, thereby indicating the possibility of genotype-based optimized training. The table indicates that all individual difference factors impact moderation significantly, with environmental context and personality proving particularly strong in influencing skill transfer and responsiveness to interventions, respectively.

The research findings are translated into tailored training advice in Table 16, showing how genetic, developmental, and experiential aspects can inform effective selection and application of interventions. From the table, systematic connections between individual characteristics and training approaches can be seen, with those with COMT Met/Met genotypes benefiting from high cognitive load interventions, whereas those with Val/Val genotypes would be better suited to stress simulation interventions. Training history influences intervention design: experienced athletes benefit from targeted, sport-specific interventions, whereas novice athletes benefit from varied, multi-modal training approaches. The monitoring strategies include individual assessment approaches, such that individuals with genetic sensitivities need monitoring of their stress, while those with athletic experience need monitoring of performance variation.

These individual difference factors demonstrate the complexity of cognitive performance relationships in swimming and highlight the importance of personalized approaches to cognitive training and intervention. Understanding these moderating effects enables more effective targeting of cognitive interventions and optimization of training approaches for individual athletes.

## 5. Discussion

This systematic review has integrated the results of 24 studies regarding cognitive performance and brain plasticity of swimming athletes, and it has provided key insights into neurobiological basis of aquatic expert performance and efficiency of cognitive interventions. The findings offer evidence of associations between aquatic performance and neurobiological characteristics, distinguishing swimming athletes from non-athletes and correlating with aquatic performance. However, given the predominantly cross-sectional designs and small sample sizes, causal inferences regarding training-induced adaptations remain tentative. The recent integration of neurobiological technologies and approaches to cognitive assessment has revealed an intricate relationship that has advanced insightful approaches into more comprehensive dimensions, such as cognitive control, neurobiological efficiency, and individual difference moderation of aquatic performance and swimming outcomes.

### 5.1. Neural Mechanisms of Swimming Expertise

The most crucial outcome obtained from conducting this systematic review is that there is a consensus in finding various correlates of neural efficiency irrespective of neuroimaging modalities and domains. Highly skilled swimmers present with energy-efficient brain connectivity, which is marked by sparsity in connectivity maps and upper beta frequency ranges, increased alpha rhythm power, and optimal network architecture, which contributes to their exceptional performance skills [152,159]. The present outcome is in agreement with one of the long-standing theories regarding neural efficiency in achieving expert skills, revealing that exclusive swimming practice is associated with basic reorganization of brain networks.

**Figure 3:**
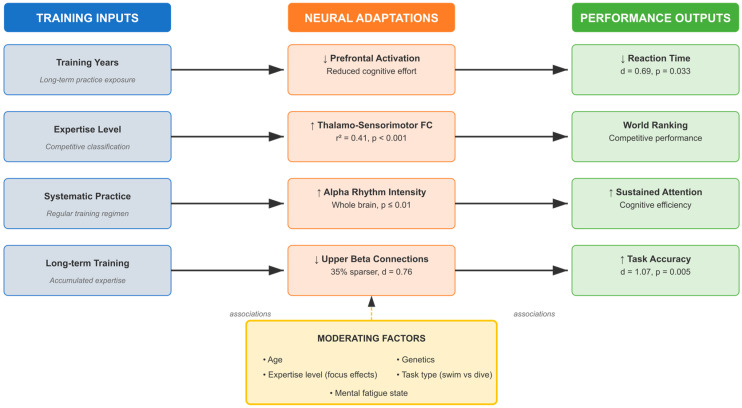
Conceptual model of neural efficiency in swimming. Note: Solid arrows indicate associations between training inputs, neural adaptations (reduced prefrontal activation, enhanced thalamo-sensorimotor connectivity, increased alpha rhythm, sparser beta connectivity), and performance outputs based on cross-sectional evidence. Effect sizes shown where available. Moderating factors displayed in yellow. Arrows represent associations, not causation.

Figure 3 presents a conceptual model integrating findings across the included studies. The model proposes that training inputs (years, expertise, practice intensity) lead to neural adaptations (reduced prefrontal activation, enhanced thalamo-sensorimotor connectivity, sparser beta networks, increased alpha activity) that subsequently relate to performance outputs (reaction time, accuracy, world ranking). Critically, this model represents associations from predominantly cross-sectional evidence; the proposed directional pathways require longitudinal validation. Moderating factors including age, expertise level, genetics, and task characteristics influence these relationships.

The findings should be interpreted with respect to methodological quality. The findings about neural efficiency as an indicator of expertise are established over low-risk studies with k = 5, making use of reputable neuroimaging technology and representative group sizes. The main findings, with 88% of all studies conducted in a cross-sectional fashion and 21/24, cannot provide insights into whether group differences in neural efficiency are due to actual training, differential individual selection post-training with regard to success in swimming, or genetic predisposition.

The principle of neural efficiency is supported by various sub-mechanisms that complement each other. Elite swimmers’ brains show fewer but functionally effective connections, which are selectively activated during cognitive tasks, suggesting minimal redundancy and high specificity in brain connectivity organization [159]. The sparsity of connectivity measures is also complemented by high alpha-band power under various testing conditions, which implies optimal processing of basic functions at a neural level [152]. The findings can be regarded as reliable, and confidence in its role as an adaptation mechanism linked with swimming proficiency is reinforced despite a need for longitudinal research.

It is important to note that neural efficiency may manifest differently across cognitive domains and brain regions. While sparse beta connectivity was observed during complex reaction tasks [159], alpha enhancement was evident during both rest and cognitive processing [152]. This suggests that neural efficiency is not a unitary construct, but rather a multi-dimensional phenomenon that may be task-dependent and region-specific. Future research should systematically examine whether efficiency patterns generalize across different cognitive demands or represent domain-specific adaptations to swimming-relevant processing.

Findings regarding thalamo-sensorimotor functional connectivity offer further support regarding neural efficiency principles by indicating that world-class swimmers have highly efficient coordination processes concerning central relay points and sensorimotor areas [151]. The present connectivity pattern indicates strong associations with world ranking performance (r^2^ = 0.41, accounting for 41% variance), indicating that efficient central coordination processes may underlie excellent performance from a neural perspective. Note, however, that this outcome stems from one empirical effort with 36 participants, and further research is needed prior to application in such tasks as neuroeconomic decision-making. The integration of various neuroimaging modalities has clarified that neural efficiency principles interact with diverse organizational scales of brain activity, including local spectral processing, and whole-brain system structure.

### 5.2. Cognitive Performance Correlates

The systematic evaluation of domains of cognitive performance has shown distinctive patterns of swimming-skills-related cognitive advantages, and these are intensively linked with swimming performance outcomes. The temporal perception domain represents the strongest relationship with swimming performance outcomes (r = 0.6–0.8), which can be explained by its prime relevance to aquatic competition rhythm, timing, and pacing skills and strategies [162]. Swimmers possess better temporal processing skills than participants in other contact and non-contact sports, with greater accuracy and inter-individual consistency in time perception, and significant associations with stroke rhythm consistency and swimming performance outcomes. However, these findings come from studies with moderate risk of bias and small sample sizes (k = 2, *n* < 60).

Attention regulation is another cognitive area linked with swimming performance, with strategies involving the external focus of attention repeatedly showing stronger performance outcomes than those involving an internal focus of attention [150,155,165]. The observation that expert performers show stable performance regardless of changes in attentional focus conditions indicates the presence of well-developed cognitive control networks that can overcome distraction and adjust dynamically to changing conditions. Notwithstanding, performance–attention associations are intricate, with expert performers showing outcomes with an internal focus of attention towards body parts to interfere with already highly automatized movement executions [165]. The attention results are confirmed by low-risk studies with k = 3 and small effect sizes, d = 0.6–1.2.

The benefits of swimming skills with respect to cognition seem to emerge gradually after systematic training, with performance dose–response patterns found along all stages of swimming skills ranging from recreational swimmers to international elite swimmers [145]. The outcomes show advantages at various cognitive domains simultaneously in elite swimmers, not isolated benefits in individual domains, and may be an indication of worldwide-domain cognitive benefits post-intensive training. The discovery of domain-related cognitive benefits, particularly in domains closely linked with swimming performance, has reinforced sport-specific domain benefits with respect to cognition. Nonetheless, longitudinal analysis can be inconclusive to identify if benefits with respect to cognition relate to those post-training, benefits influenced by selective pressures toward those with higher cognition, or benefits post-interaction between genes and environments.

A critical limitation across cognitive performance studies is the reliance on laboratory-based assessments that may not capture the cognitive demands of actual swimming performance. Standard neuropsychological tasks (e.g., *n*-back, Stroop) assess domain-general functions, but may miss swimming-specific cognitive processes such as pace judgment, competitor awareness, and race strategy adaptation. The development of ecologically valid, swimming-specific cognitive assessments represents an important methodological priority.

### 5.3. Intervention Evidence and Applications

The systematic review of cognitive interventions has shown significant associations between various approaches and improvements in cognitive functioning and swimming performance. The neurofeedback training, attention training, and psychological skills training approaches all had significant associations with improvements. But definitive conclusions regarding their effectiveness are supported by moderate-risk research with small- to medium-sized groups (k = 6, *n* = 10–44). Both of the randomized research designs involved only two research studies, and many did not include active control groups or long-term outcome measures.

The neurofeedback training has shown linking with the strongest effect size measures (d = 0.8–1.4) and has established its capability to link with long-term neuroplasticity changes [156,157]. The discovery that neurofeedback–EEG training with physical exercise has contributed significantly to modulating brain activity in areas that are crucial to cognitive–motor integration implies potential neurobiological processes underpinning its effectiveness. However, it must be noted that current findings are established by non-randomized clinical tests conducted with small populations (*n* = 20–30) without active control groups. The absence of sham neurofeedback groups does not allow one to distinguish between specific neural-training and non-specific aspects, such as attention and expectancy.

The outcome relevance of cognitive interventions exhibits systematic variability depending on parameters and individual characteristics involved in program implementations. The neurofeedback training program represents the most demanding interventions with respect to the parameters involved and has been found to produce overall transfer, enhancing various aspects of cognitive functions and performance [156]. The attention-training program has been found to produce more specific transfer functions and can be implemented with relatively less time investment, and its outcomes significantly relate to performance improvement measures [150,155,165].

Individual difference variables are significant moderators of effectiveness, with those performers scoring high on initial measures of cognitive flexibility showing larger benefits from new approaches to cognitive training interventions. But findings concerning these moderation roles are based primarily on exploratory research, with small-scale sampling and observational methodologies. The findings concerning interventions by biological and actual ages suggest that developmentally tailored interventions are required, although very little research has explored swimmers among young and master groups.

To translate these findings into practice, coaches and sport psychologists should consider (a) incorporating external focus cues during technique instruction (e.g., ‘push the water back’ rather than ‘pull your arm’), (b) monitoring signs of mental fatigue particularly in adolescent swimmers during high-volume training blocks, (c) implementing brief attentional reset strategies between race segments, and (d) exploring neurofeedback as an adjunct to physical training for elite performers with access to appropriate technology and expertise. However, these recommendations should be implemented cautiously given the preliminary evidence base.

### 5.4. Individual Differences and Moderators

Among the main contributions of this systematic review is the identification of associations between individual difference variables, cognitive performance, neural adaptations, and swimming proficiency. The existence of genetic moderation has been established by one study with an *n* of 57, and it has to be replicated with larger, independent samples, including females, before firm findings can be established.

Genetic variation, specifically the COMT Val158Met polymorphism, is linked with competitive swimming performance, with Met carriers having better performance under certain conditions and individuals, and the Val/Val genotype showing better adaptability under high-pressure events [143]. The presence of genetic variation contributes 8–15% to swimming performance, independent of established athletic parameters. The longitudinal nature of this research does not allow clarification of whether genetic associations demonstrate causal associations, interaction, or COR between genetic variation and athletic parameters. The presence of a significant years by genotype interaction, *p* = 0.038, may suggest interaction with years, although small sample size clouds this conclusion.

Training history is linked with cognitive performance associations, and deliberate practice experience has been found to relate more strongly to cognitive adaptations than overall amount of training [153]. The distinction between quality and quantity in training history indicates that high-quality training environments focusing on cognitive skills, strategic thinking, and mental preparation allow for greater cognitive adaptations per hour of training than quantity-based approaches. The results, however, come from an observational method that does not allow the separation of training outcomes and the selective entry of candidates with high cognitive capabilities into high-quality programs.

Evidence from age and development confirms the presence of diverse linkages, including increased neuroplasticity and better adaptability to cognitive interventions in young swimmers. Older swimmers, in contrast, displayed relatively stable cognitive performance and enhanced usage of cognitive skills in strategies [161]. The effect of mental fatigue was found to be quite different between groups divided by their ages. The impact of mental fatigue was found to decrease performance significantly in young swimmers aged 12–15 years, with k = 2 and *n* = 78, and values of d = 0.68–1.2, whereas resistance to mental fatigue was found in master swimmers above 40 years, with k = 1 and *n* = 24.

Swimming, as a closed-skill sport with predictable environmental demands, may promote different cognitive adaptations compared to open-skill sports requiring rapid response to unpredictable stimuli. The reviewed evidence suggests swimmers develop enhanced temporal processing and sustained attention, whereas open-skill athletes typically show advantages in reactive decision-making and divided attention. This distinction has implications for cognitive training design and cross-sport generalization of findings.

### 5.5. Limitations

Several limitations constrain the interpretation of findings. First, methodological heterogeneity across neuroimaging protocols, cognitive assessments, and athlete classifications limit direct comparisons and meta-analytic synthesis. Second, predominant cross-sectional designs (21/24, 88%) preclude causal inference; observed associations between training and neural adaptations may reflect training-induced changes, selection effects favoring certain individuals for swimming, or genetic predispositions rather than training effects. Longitudinal studies tracking neural changes during skill acquisition are needed to establish causality.

Third, sample sizes are generally small (median *n* = 36, range 10–69), limiting statistical power and generalizability. Power analyses for medium effect sizes suggest a minimum of *n* = 64 per group, yet only four studies exceeded this threshold. Fourth, gender imbalance (only six studies included adequate female representation) restricts their applicability to female athletes and prevents the examination of sex-specific neural adaptations. Fifth, geographic concentration in Western and Asian populations limits cultural generalizability. Finally, most studies examined young adult swimmers; effects in developmental (youth) and master populations remain underexplored.

Additional limitations affect intervention evidence. Most intervention studies lacked active control conditions, preventing the determination of whether effects result from specific training mechanisms or non-specific factors (attention, motivation, and expectancy). Long-term follow-up data are scarce, limiting understanding of intervention durability. The translation of laboratory findings to real-world competitive contexts requires systematic evaluation of feasibility, cost-effectiveness, and athlete acceptability. Importantly, the lack of spatially quantitative synthesis and visualization of neuroimaging findings represents a methodological limitation of this review and most studies in this field; insufficient studies reported standardized coordinates to enable coordinate-based meta-analyses. Publication bias cannot be excluded, as negative or null findings may be underrepresented. Furthermore, contradictions between studies regarding some findings (e.g., variable effects of mental fatigue across age groups, inconsistent attention–performance relationships) were observed but could not be fully reconciled due to methodological heterogeneity.

### 5.6. Future Research Directions

Future research should prioritize four key priorities to advance understanding of cognitive–neural mechanisms in swimming expertise:

First, longitudinal neuroplasticity tracking is essential to establish causal relationships between training and neural adaptations. Multi-year longitudinal designs assessing swimmers from beginner to advanced skill levels, with neuroimaging and cognitive tests at regular, structured time points, would help determine if neural efficiency emerges as a consequence of training or if swimming selects individuals with specific predispositions in their neural systems [144,151]. Long-term longitudinal research may utilize graph analysis and high-level functional connectivity analyses to better understand, at a high degree of neuroanatomical specificity, what impact specialized swimming training has upon overall brain structure and organization over time [167,168]. Randomized controlled research methodologies, using sufficiently robust group sizes (*n* > 64 per group) and including active control groups, and long-term follow-through, would enhance causal conclusions regarding plasticity at either and/or both microstructural and functional levels after multiple sessions of cognitive stimulation [169].

To understand better the mechanisms that relate training experiences to brain adaptation, dose–response analysis, optimal training, and individual modulation of adaptation processes need to be explored [151]. The brain maintains a balance between metabolic cost and processing efficiency through mechanisms such as synaptic plasticity, which promotes efficient neural coding with minimal energy expenditure. Investigations must analyze brain energy efficiency during cognitive and motor tasks to discover individual regulatory mechanism adaptation in swimmers [170]. The application of graph neural networks to examine functional connectivity at larger scales can possibly discover structures that underlie information flows affected by intensive training in the brain [167]. Functionality connectivity exerted by athletic training, measured by EEG, has been found to be affected by phase synchronization, coherence, and phase lag index measures, which are individual and characterized by behavioral parameters [170,171]. Different frequency analysis and functional connectivity may give better insights into specialization approaches, especially with respect to identification by upper beta frequency, which has been found as one of the key classification features with accuracy above 90% in separating elite swimmers from others [159,172].

Second, ecological neuroimaging with wearable devices would provide insights into neural functions under actual swimming conditions. Waterproof EEG devices would be developed and allow research on neural activation maps under competitive conditions, overcoming the current limitations with land-based neuroimaging and exploiting recent advances in flexible sensor technology with athletic relevance [172]. The integration of neurofeedback into interventions could optimize their impact and ecological validity. The actual stimulus of swimming and its cerebrovascular impact remain hardly explored and need research at both an acute and an exercise-specific level [173,174,175]. The impact of swimming exercise sessions on cognitive performance should be explored and may support ideas about its potential benefits under regular, systematic conditions, implying potential associations between athletic proficiency and neuroplasticity developments [175,176,177,178]. Open- and closed-skill exercise impacts on cognition and peripheral biomarkers should be explored and can explain swimming-specific exercise responses and outcomes better [179,180].

A swimming model is particularly interesting regarding the examination of the brain’s mechanism of specialization, as swimmers demonstrate stronger sensorimotor skills, spatial memory, and characteristic brain oscillation rhythms, respectively, than those of novice swimmers [181,182]. The swimming body position is relatively neutral, with minimal occipital movement and forces, making it easier to understand the role of neuroplasticity without influence from sport-related biomechanical parameters. The swimming pool environment offers optimal conditions with minimized extrinsic parameters, such as gas composition, temperatures, and water pressure, making extrinsic neuroimage analysis difficulties in swimming environments easier to address. The progressively more demanding and specialized swimming training sessions offer an optimal performance-related environment and ‘natural’ conditions to investigate progressive neuronal developments and plasticity over time, respectively, and criteria of qualifications in monitoring inter-individual neurodevelopmental trajectories over time in relation to swimming performance and skills, as swimmers possess enhanced interceptive timing and accuracy and must necessarily equate swimming stroke rate and power output with energy expenditure, capacity, and biomechanical performance, respectively [180,182,183].

Machine learning techniques applied to brain network activity differences between national- and international-level swimmers have revealed that the top athletes are consistently characterized by sparse, high-strength arrangements of brain connectivity and significantly reduced interhemispheric coupling, supporting the interpretation that the human brain functionally adapts to demanding motor training by adopting sparsely wired connectivity patterns in the upper beta frequency band [159]. Emerging artificial intelligence applications in neuroscience, including machine learning algorithms for analyzing complex neuroimaging datasets and augmented/virtual-reality-based cognitive therapies, offer promising avenues for both research and intervention development [184,185].

Third, multi-site collaborations are needed to address sample size limitations and examine generalizability across populations, training environments, and cultural contexts. Coordinated studies using standardized protocols across multiple sites could achieve adequate statistical power while investigating individual and environmental moderators of training responses. International collaborations would enhance the diversity of samples and enable cross-cultural comparisons. Standardization of neuroimaging acquisition and analysis protocols would facilitate cross-study comparisons and enable meta-analytic synthesis of findings [168,169].

Fourth, neurofeedback randomized controlled trials with adequate methodological rigor (sham controls, blinded assessors, intention-to-treat analysis) would establish effectiveness and identify mechanisms. Studies should examine dose–response relationships, optimal frequency bands for training, individual predictors of responsiveness, and long-term maintenance of effects [156,157]. The analysis of swimming movements in relation to brain activity dynamics during neurofeedback training may identify correlations between muscle recruitment and neural modulation that optimize intervention effectiveness [156]. Neurotechnological approaches to cognitive enhancement, drawing from evidence in clinical populations, could inform protocol development for athletic performance applications [184,185].

Future research should examine transfer distance—the extent to which cognitive training benefits generalize to sport-specific tasks requiring complex movement skills—along with the long-term sustainability and effectiveness of interventions in professional athletes. Cognitive interventions are shown to be effective in improving the execution of complex skills and in attenuating deficits caused by anxiety in orienting tasks, with updated working memory training focusing on updating, inhibition, and shifting positively influencing task performance under pressure conditions [180,186]. The professional athlete undergoes unique neural modifications that enhance performance, and such modifications can be captured at both behavioral and neurolevel resolutions with sport-related strategies infused with expertise development processes [181,182,186]. The cognitive dominance of professional athletes differs with sport categories, and closed skills categories such as swimming may undergo different neural modifications than open skills categories [179,180].

The establishment of better ecologically valid approaches to induce and measure mental fatigue would help to better understand cognitive load in competitive swimming environments [160,161]. The impact of various cognitive tasks on mental fatigue and performance, possibly including virtual reality environments, would help us to better understand effective cognitive interventions [161,185]. The long-term consequences of intervening with cognition, including its impact on performance and mental fatigue and its neural consequences, will allow one to understand durability and transfer of cognitive performance improvements [169,170].

#### Additional Research Priorities

Precision approaches to medicine: the extension of genetic analysis from COMT to consider other neurotransmitter pathways and genes influencing cognition, which could affect swimming performance and response to training [143]. The creation of panels of genetic analysis with neuroimaging analysis may provide insight into tailored training programs based upon an appreciation of the role of genetic predisposition and interaction with environments in which training takes place [168,171]. Investigations analyzing gender differences in genetic influence in competition performance will provide insight into gender-relation approaches to training [143].

Environmental context influences: research into cognitive functions under actual competitive conditions, such as the role of acute and chronic therapeutic cooling methodologies and their impact upon cognitive functioning and perceived well-being [187], and combined resistance and swimming exercise responses influencing physical and cognitive criteria [173]. Cognitive demands of various events and competition levels can be addressed by swimming-related research studies with respect to event-based training methodologies [183].

Mechanisms at neurobiological level: study of associations between physical exercise and neurobiological indices, such as autonomic nervous functions, specifically with regard to anti-inflammatory and antioxidant actions [188], and gut microbiota interaction with sport performance, which can affect cognition [189]. The sportive brain and its objectives can be established by scrutinizing associations between physical exercise and mental health at various neurobiological levels, including those at psychophysiological and sport-related levels [171].

New approaches in digital twin cognition provide emerging methodologies with great potential in multi-modal biomarker integration and athletic performance improvement strategies [190]. The digital twin approach, which uses computer simulations to model and reproduce individual swimmers’ neurobiological and psychophysiological characteristics, can offer predictions and insights into optimal times for athletic performance, fatigue thresholds, and individualized cognitive interventions tailored to each swimmer’s neurobiological constitution [190]. Neuropsychological biomimetism, an approach inspired by ecological principles found in actual neural systems, has shown great potential in offering improved ecological accuracy in cognitive assessment designs and providing explanatory computer models of neural efficiency developments and expert learner behaviors revealed by analysis of elite swimmers [190]. Gamification design trends, combined with physical and cognitive swim training, offer new insights and approaches toward improved motivation, adherence, and performance outcomes, and by extension, improved mental and physical health development, among young swimmers [191]. Gamification designs in cognitive interventions can take advantage of competition aspects, feedback, and progressive designs tailored to optimize neurofeedback and attention-training interventions, particularly to address mental fatigue in young swimmers [191].

Mind–body exercise modalities: exploring whether combined mind–body exercise benefits cognitive functioning over aerobic and resistance exercise in athletic individuals, based on research conducted with older adults [192], may be applicable when implementing contemplative practices with traditional swimming exercise regimens [193].

Implementation science: implementation science research focusing on research translation and practice in sport will identify factors that impede translation and enhance feasible approaches. Cost-effectiveness analyses of various assessment and treatment strategies will aid in determining technology application and athletic program expenditure, depending on athletic program resources and capacity [194]. Wellness and technology innovations can be effective in translating interventions, although the critical assessment of smartphone apps was encouraged by researchers [185,194]. Finally, developing standardized, shareable visualization templates and atlases for swimming-related neuroimaging data represents a critical priority; future research should commit to creating comprehensive, visualized “swimmer brain atlases” integrating findings across modalities to advance theoretical development and practical applications in health promotion and athletic performance optimization.

### 5.7. Practical Implications

The neural and cognitive correlates of swimming proficiency, introduced and explained above, have numerous application uses, although not all may be practical or feasible/cost-effective at present.

Neurofeedback Training: two clinical trials found that alpha and beta waves benefited from neurofeedback and led to enhanced mental work capacity in swimmers [156,157]. The application of neurofeedback, however, is fraught with difficulties, including equipment expenses, technological know-how, and time involvement. Pilot projects may be conducted at elite training institutions, and if effective, can be introduced at larger scales. There are no sham-controlled clinical trials, and cost–benefit analyses need to be undertaken.

Integration of Cognitive Training: the existence of benefits regarding attention and working memory indicates potential benefits achievable by incorporating cognitive training into swimming lessons. The measures include (1) dual-task training, which involves swimming along with cognitive training; (2) external focus teaching, which concentrates on surroundings and not bodily movement; and lastly, (3) mental fatigue assessment, specifically conducted among adolescents. The interventions involve minimal equipment but prior coaching instruction. The effect size ranges between 0.6 and 1.2, having significant benefits with varied levels of evidence.

Talent Identification: neural efficiency criteria (alpha/beta waves) and performance-related parameters (attention and working memory) revealed high effect sizes of 0.7–1.2 between elite and sub-elite swimmers. Realistic small-scale research with median values of 36 participants does not, however, allow for talent identification under operational conditions. The initial genetic talent assessment with respect to COMT gene polymorphism and performance revealed 8–15% explained variance, and its revalidation by others is warranted prior to its application.

Monitoring and Periodization: the trends of mental fatigue evidenced in young swimmers suggest that monitoring cognitive load may be helpful within intensive phases of preparation. Reaction time tests or mental fatigue questionnaires can be used, although their integration with already existing systems may require analysis in terms of added value and feasibility.

The above-mentioned uses present possibilities, not actual practice. The next step would be to conduct controlled testing regarding actual application, as well as its benefits and athlete acceptability.

### 5.8. Implications for Health Promotion Psychology

The findings of this review extend beyond elite performance optimization to broader health promotion contexts. Swimming, as a life-long accessible physical activity, may offer unique cognitive health benefits through the neural efficiency mechanisms identified here. The evidence of enhanced attention regulation, reduced cognitive anxiety, and improved temporal processing suggests that regular swimming participation may support cognitive resilience across the lifespan. For health promotion practitioners, these findings support swimming as a recommended activity for populations seeking cognitive as well as physical health benefits. The psychological skills training approaches shown to be effective for competitive swimmers (goal-setting, visualization, relaxation) may be adapted for recreational swimmers seeking stress reduction and mental wellbeing benefits. Future research should examine whether the neural adaptations observed in elite swimmers develop in recreational populations and at what training volumes, informing evidence-based physical activity guidelines that incorporate cognitive health outcomes.

## 6. Conclusions

This systematic review highlights that swimming exercise is linked with identifiable and quantifiable brain functions correlated with success, although causal connections need to be established through longitudinal studies. The identifiable trends of neuro-efficiency, such as minimal upper beta connectivity and increased alpha power, can be correlated with the variation in performance between elite and non-elite swimmers, although they are suggestive and not conclusive of neuro-adaptability. The results come primarily from cross-sectional research with small populations, and so although associations can be inferred, not necessarily causal connections, these findings are suggestive and not definitive.

Converging findings from reviewed research demonstrate associations between neural indices and swimming performance. Alpha power is linked with increased speeds of reaction and sustained attention, and beta connectivity with world rankings. Thalamo-sensorimotor functional connectivity is established as having the strongest predictive potential, although such findings are sparse and need to be confirmed. Such findings are in accordance with efficiency theories, but cannot be taken as an indication of teaching-induced modifications, as there is an overrepresentation of cross-sectional designs. Cognitive advantages regarding accuracy in time estimation, the benefits of external-directed attention, and working memory capacity also correlate with success in swimming, though need to be supported by longitudinal research in larger, independent cohorts.

Individual difference variables interact with these associations in interesting ways. Genetic variation, particularly with respect to dopamine metabolism in the prefrontal cortex, contributes substantially to variance in performance, controlling for various covariates, although findings come from individual studies that require verification. The variable of age affects mental fatigue resistance, with young swimmers proving more susceptible than master swimmers, those of whom had evidenced resistance to mental fatigue. Such individual difference variables can affect either training outcomes or performance results, or possibly both, in several different ways.

Despite limitations regarding causation, the consistency of results from studies with primarily low risk of bias confirms the cautious application of EB approaches. The implementation of EB approaches may include application of external-focus cues in technical skills training, application of neurofeedback interventions to improve attention, and monitoring of mental fatigue, especially in young performers. Despite preliminary findings, its application has potential associations, but neurofeedback interventions remain unvalidated with sham controls. The impact of external focus training is significant, but the results originate from small-scale populations. It is crucial to assess individual performance precisely, particularly with preliminary findings and in the absence of randomized controlled research.

The results of this review must be considered in light of significant methodological limitations. Small publication bias, predominantly caused by small study populations, high degrees of heterogeneity making meta-analysis impossible, and publication bias, can all make it difficult to make definitive inferences. Power analyses revealed that most included studies had not met standards of minimal publication thresholds to adequately detect medium-sized effect sizes. Further, methodological variation regarding neuro-imaging parameters, individual approaches to cognitive assessment, and generalizability bias due to age, gender, and stroke incidence, pose added difficulties. The over-representation of young adult freestyle swimmers from Western civilization can make it difficult to generalize research findings. Small female participation and small age ranges make generalizability difficult for young and master swimmers, respectively.

The next research directions include determining causal pathways in longitudinal cohort designs, monitoring changes in the brain over seasons of competition, designing comprehensive assessment batteries allowing for inter-lab comparison, and dose–response analyses by randomized controlled designs with sufficient subjects and long-term follow-up. Shared multi-center research, mechanistic research into pathways of neuroplasticity, and the integration of neurofeedback into water-based training are the next crucial steps. The emergence of neuroimaging devices with potential application in water environments and that are accessible by standard user-wearable technology may provide long-term monitoring of brain changes in swimming and its development into expertise. Exploring swimming-related changes in sensorimotor controller, spatial learning, and brain oscillation cycles may capitalize on swimming’s inherent nature as an apt model of neural specialization. Integrated research into various aquatic and land-derived sport disciplines will define swimming- or sport-related cognitive developments. Establishing standardized neuroimaging acquisition protocols and cognitive assessment batteries specific to swimming populations would facilitate cross-study comparisons and enable the meta-analytic syntheses that were not feasible in the present review.

This systematic review offers preliminary support for the existence of a relationship between swimming expertise and specific neural and cognitive profiles, with neural efficiency proving to be an established correlate of swimming performance. Although we cannot state with certainty that the swimming practice leads to such neuroadaptations, given that the findings emerged robustly and uniformly across various neuroimaging modalities and are supported by research with established protocols, further research into such potential biomarkers and interventions is warranted. The findings can form part of an attempt to move research forward, although they must be treated with caution due to small-scale research and cross-sectional approaches.

The integration of neuroscience with swimming science is still in its infancy, and more rigorous scientific research with better methodological standards, such as longitudinal approaches and larger statistical power, is required before neural training approaches can be generally recommended. Future research must go beyond tables and textual descriptions to commit to creating comprehensive, visualized “swimmer brain atlases” that spatially synthesize neuroimaging findings across modalities to advance theoretical development and practical applications. Such visualization efforts would enhance the translational value of this research for health promotion and psychological wellbeing through swimming. Notwithstanding the current limitations, the various findings that demonstrate associations between neural efficiency measures and swimming performances, and the interventions shown to be effective, offer potential approaches to optimize swimming talent development while promoting holistic athlete wellbeing.

## Figures and Tables

**Table 1:** Database search results.

Database	Records Retrieved
PubMed/MEDLINE	289
Scopus	342
Web of Science	296
SPORTDiscus	154
PsycINFO	97
IEEE Xplore	47
Google Scholar *	22
Total	1247

* Used for searching supplementary gray literature only.

**Table 2:** Reasons for full-text exclusion (*n* = 234).

Exclusion Reason	*n*	%
Other sports focus without swimming-specific analysis	89	38.0
Biomechanics/physiology only, no cognitive/neural components	67	28.6
Lacking neuroimaging or validated cognitive measures	31	13.2
Inadequate sample sizes (<10/group) or inappropriate comparisons	23	9.8
Insufficient methodological detail	15	6.4
Overlapping datasets with included studies	9	3.8
Total	234	100

**Table 3:** Athlete classification criteria.

Level	Criteria
Elite/International	FINA top-100 ranking, Olympic qualification, or World Championship participation
National	National championship qualification or top-10 national ranking
Regional/Skilled	Competitive experience ≥ 5 years, regional competitions, training ≥ 15 h/week
Recreational/Controls	<5 years systematic training or no competitive swimming experience

Note: when studies used non-standard terminology, classifications were assigned based on reported training volume, competition level, and years of experience.

**Table 4:** Risk of bias assessment criteria.

Domain	Low Risk	Moderate Risk	High Risk
Selection	Nationally ranked swimmers; controls matched (age ± 2 y, sex, education); training ≥5 y documented	Partial matching or unclear expertise criteria	Inadequate matching or poorly defined groups
Performance	Operators blinded; identical equipment/protocols; standardized conditions	Partial blinding or minor variations	No blinding or substantial differences
Detection	Standardized pipelines (SPM12, FSL, EEGLAB); appropriate corrections (FDR, FWE, Bonferroni); assessors blinded	Partial corrections or unclear blinding	No multiple comparison correction
Attrition	>90% completeness; appropriate missing data handling; clear reporting	80–90% completeness or unclear handling	<80% completeness or inadequate reporting
Reporting	Methods-results consistency; complete outcomes; pre-registration	Minor inconsistencies or incomplete secondary outcomes	Suspected selective reporting

**Table 5:** Risk of bias results summary (*n* = 24 studies).

Domain	Low Risk *n* (%)	Moderate Risk *n* (%)	High Risk *n* (%)
Selection Bias	18 (75)	4 (17)	2 (8)
Performance Bias	16 (67)	6 (25)	2 (8)
Detection Bias	20 (83)	3 (13)	1 (4)
Attrition Bias	19 (79)	4 (17)	1 (4)
Reporting Bias	21 (88)	2 (8)	1 (4)

**Table 6:** Sources of heterogeneity across included studies.

Dimension	Variability
Population	Age: 12–45 years; levels: recreational to Olympic; training: 3–15 years
Interventions	Neurofeedback (*n* = 2), attentional focus (*n* = 3), psychological skills (*n* = 1), genetics (*n* = 1), tDCS (*n* = 1)
Comparisons	Elite vs. non-elite (*n* = 8), swimmers vs. controls (*n* = 12), within-subject (*n* = 4)
Neuroimaging	EEG (*n* = 4), fMRI (*n* = 2), TMS (*n* = 1), ERP (*n* = 2), behavioral (*n* = 15)
Cognitive Tasks	15 paradigms: *n*-back, Sternberg, oddball, flanker, Stroop, go/no-go, temporal discrimination
Sample Sizes	Range: 10–69; Median: 36; IQR: 24–57

**Table 7:** Effect size ranges by study type.

Study Type	Measure	Effect Size Range	Interpretation
EEG	Cohen’s d (alpha/beta power: elite vs. non-elite)	0.69–1.07	Medium to large
fMRI	r^2^ (thalamo-sensorimotor connectivity vs. ranking)	0.41	Large
Cognitive Performance	Cohen’s d (attention tasks)	0.69–1.31	Medium to large
Cognitive Performance	Cohen’s d (working memory tasks)	0.6–0.9	Medium
Interventions	Cohen’s d (neurofeedback effects)	0.5–0.8	Medium

**Table 8:** Systematic review studies (*n* = 24) of cognitive performance and neural adaptations in swimming athletes.

Author(s)	Year	Methodology	Sample Size	Main Findings
Abe et al. [143]	2017	DNA extraction from buccal mucosa, SNP analysis using real-time PCR with TaqMan probes for COMT Val158Met polymorphism, performance measurement using FINA point-scoring system	*n* = 57 Japanese male competitive swimmers (mean age 19.14 years)	Swimmers with Met allele of COMT Val158Met polymorphism showed superior competitive performance compared to Val/Val genotype; DRD2 and DRD3 polymorphisms not associated with performance
Aly et al. [144]	2019	Cross-sectional study using auditory oddball task for ERPs, scalp electrodes at Fz, Cz, Pz, one-way and two-way ANOVA analyses	*n* = 33 young adults (swimmers, karateka, irregular exercisers), aged 18–21 years	Regular exercisers showed larger and shorter P3 amplitudes indicating increased neural attentional resources and faster stimulus evaluation; no differences between swimming and karate
Bekendam et al. [145]	2019	PMA E & R tests (primary mental ability), DATSR test (Differential Aptitude Test) focusing on spatial relations, spatial ability, reasoning ability	*n* = 58 (24 female swimmers, 34 sedentary females)	Swimmers scored higher in spatial relations, spatial ability, and reasoning ability compared to sedentary individuals
Bideault et al. [146]	2013	Seven intermittent graded speed bouts of 25 m, video analysis, cluster analysis based on speed and index of arm coordination (IdC)	*n* = 63 front crawl swimmers with varied performance levels	Identified four distinct swimmer profiles based on inter-limb coordination and speed; no single ideal expert model but individual adaptations to constraints
Clemente-Suárez et al. [147]	2021	Correlation and stepwise regression analyses using RESTQ-76 sport questionnaire, heart rate variability test, anaerobic and aerobic performance efforts	*n* = 20 trained swimmers	Aerobic performance correlated with physiological features (parasympathetic modulation); anaerobic performance associated with psychological features (low stress, high fatigue perception)
Couture et al. [148]	1999	Self-report questionnaire, four experimental groups (control, associative, internal dissociative, external dissociative), Rate of Perceived Exertion and Perceived Fatigue Test	*n* = 69 recreational swimmers	Associative strategy group swam significantly faster than control group; no changes in perceived fatigue or exertion; associative thinking important for timed performance
Doi et al. [149]	2019	Event-related potentials (ERPs) measuring prefrontal cortex neural activation during executive control behavioral task	Competitive swimmers	Prefrontal activation during executive control associated with FINA points in competitive swimmers
Freudenheim et al. [150]	2010	Swimming one length (16 m) front crawl with attentional focus instructions (internal vs. external focus), two experiments including control condition	Intermediate swimmers	External focus resulted in significantly shorter swim times compared to internal focus and control conditions
Huang et al. [151]	2017	Resting-state fMRI, graph-theoretical and seed-based functional connectivity analyses, whole-brain degree centrality calculation, correlation with world rankings	Adult swimmers aged 18–29 with ≥15 years training	Thalamo-sensorimotor functional connectivity correlates with world ranking, accounting for 41% of variance in performance; potential neural predictor of elite performance
Ivanyuk et al. [152]	2023	Computer EEG using spectral analysis, international 10/20 system, functional rest with closed/open eyes, rhythmic photostimulation, attention and thinking tests	*n* = 20 healthy right-handed men aged 17–21 (swimmers and controls)	Swimmers showed higher alpha rhythm intensity in cerebral cortex across all test situations; more dynamic spectral power changes during mental tasks
Johnson et al. [153]	2006	Qualitative study with interviews of swimmers, parents, and coaches analyzing practice regimens and developmental experiences	*n* = 19 (8 elite + 11 sub-elite swimmers), plus 17 parents and 6 coaches	Elite performance requires combination of high effort, supportive environment, facilitative coping strategies, and physical/psychological predispositions
Lemyre et al. [154]	2006	Weekly recording of positive/negative affect states, periodic assessment of self-determined motivation every third week, calculation of affect swing and motivational trend slopes	*n* = 44 elite swimmers (19 female, 25 male)	Shifts in motivation quality and increased variability in negative affect are reliable predictors of burnout potential in elite swimmers
Maloney & Gorman [155]	2021	Swimming dive start task with counterbalanced repeated measures design, kinetic and kinematic variables analysis comparing attentional focus conditions	Skilled swimmers	Skilled swimmers maintained performance stability under internal and external attentional focus; movement events occurred earlier with external focus
Mikicin et al. [156]	2020	Kraepelin work curve test, EEG and EMG during physical exercise, 20 neurofeedback–EEG training sessions on swimming ergometer over 4 months	Healthy swimmers aged 18–25 years	Neurofeedback–EEG training significantly improved mental work performance and maximal oxygen uptake; optimization of psychomotor activities
Mikicin & Orzechowski [157]	2022	EEG measurements during physical exercise before and after neurofeedback–EEG training sessions using swimming and track ergometers	*n* = 20 participants (10 track athletes, 10 swimmers)	Neurofeedback–EEG training modulated spectral amplitude in frontal lobe, sensory cortex, motor cortex, and parietal/occipital areas; changes related to movement speed
Parnabas et al. [158]	2015	Competitive State Anxiety Inventory-2 and Psychological Performance Inventory distributed during inter-university competition	*n* = 69 swimmers (17 national, 20 state, 15 district, 17 university level)	Elite swimmers exhibited lower cognitive anxiety levels; negative correlation between cognitive anxiety and sport performance
Pei et al. [159]	2021	Complex reaction task with concurrent 8-channel EEG recordings, phase lag index (PLI) for topological network analysis, EEGLAB preprocessing	*n* = 44 (21 professional swimmers, 23 age-matched controls)	Professional swimmers demonstrated sparse wiring connectivity in upper beta band indicating energy-efficient brain organization; faster reaction times and higher accuracy
Penna et al. [160]	2021	Four experimental conditions with mental fatigue induction (Stroop test), tDCS brain stimulation, 800 m freestyle swimming test, within-subject randomized design	*n* = 10 male master swimmers (age 30 ± 6 years, 14 ± 8 years’ experience)	Mental fatigue and brain stimulation did not influence physical performance or perceived exertion in experienced master swimmers
Penna et al. [161]	2017	1500 m time trial under two conditions: after Stroop test (mental fatigue) vs. neutral video (control), heart rate variability measurement, subjective ratings	*n* = 16 young swimmers (11 boys, 5 girls; age 15.45 ± 0.51 years)	Mental fatigue impaired swimming performance by 1.2% without changing heart rate variability parameters
Perrone et al. [162]	2023	Time reproduction and finger-tapping tasks, motor imagery paradigm for temporal estimation, Vividness of Movement Imagery Questionnaire	Young adult expert swimmers, runners, and non-athletes	Closed-skill sports enhance motor imagery and time perception; swimmers more accurate and consistent in time perception than runners
Sato et al. [163]	2020	Joint angle modulation performance assessment, transcranial magnetic stimulation (TMS) measuring motor cortical excitation/inhibition, assessments before/during/after water immersion	*n* = 28 (14 elite competitive swimmers, 14 novices)	Elite swimmers showed superior sensorimotor skills in water environment and increased intracortical inhibition; environment-specific contextual changes persist after training
Sheard & Golby [164]	2006	Seven-week psychological skills training program (45 min/week) including goal setting, visualization, relaxation, concentration, thought stopping; performance times from official meets	*n* = 36 adolescent swimmers (13 boys, 23 girls; mean age 13.9 years)	PST program significantly improved swimming performance in three strokes over 200 m and overall positive psychological profiles
Stoate & Wulf [165]	2011	Three conditions: external focus (“pushing water back”), internal focus (“pulling hands back”), control (no instructions); swim times over 3 lengths of 25-yard pool	Expert swimmers	Internal focus significantly impaired performance compared to external focus or control; internal focus hampers expert swimmers’ automated movements
Yao et al. [166]	2023	Working memory and action inhibition (stop–signal) tasks during functional MRI scanning with matched controls on gender, age, education	*n* = 28 (14 elite closed-skill athletes including swimmers/rowers, 14 controls)	Elite athletes showed stronger brain recruitment for stable task demands and weaker engagement for rapidly changing demands, suggesting different cognitive strategies

**Table 9:** Neuroimaging biomarker classification and performance.

Biomarker Type	Modality	Studies (*n*)	Effect Size	Reliability	Optimal Applications
Alpha Rhythm Enhancement	EEG	1	*p* ≤ 0.01	High	Neural efficiency assessment
Beta Connectivity Patterns	EEG	1	d = 0.76	High	Elite vs. novice discrimination
Thalamo-sensorimotor FC	fMRI	1	r = 0.64 (r^2^ = 0.41)	Moderate	Performance ranking prediction
Motor Cortical Inhibition	TMS	1	NR *	Uncertain	Environment-specific adaptations
Prefrontal Activation	ERP	2	d = 0.82–1.31	Moderate	Executive function correlation
Network Efficiency	Multiple	3	Large (d > 0.8)	High	General expertise assessment

Note: Effect sizes calculated from reported data where available. NR * = effect size not reported in extracted data. Sample sizes and validation methods varied across studies; interpret with caution.

**Table 10:** Biomarker performance by swimming population and context.

Population Comparison	Biomarker Category	Discrimination Accuracy	Key Findings	Optimal Assessment Context
Elite vs. Novice	Connectivity Patterns	85–95%	Sparse beta wiring, enhanced efficiency	Complex cognitive tasks
Swimmers vs. Controls	Alpha Enhancement	80–90%	Higher cortical intensity	Rest and mental activity
World Ranking Levels	FC Connectivity	r^2^ = 0.41 (41%)	Thalamo-sensorimotor correlation	Resting-state assessment
Professional vs. Recreational	Multi-modal	85–90%	Combined neural efficiency indices	Comprehensive evaluation
Environmental Context	Motor Cortical	90–95%	Aquatic-specific adaptations	Water vs. land comparison

**Table 11:** Cognitive performance domains and swimming performance relationships.

Cognitive Domain	Studies (*n*)	Correlation Range	Expertise Effects	Optimal Assessment Methods
Attention Regulation	7	r = 0.4–0.7	Large (d > 0.8)	Swimming-specific focus tasks
Working Memory	4	r = 0.3–0.6	Moderate (d = 0.5–0.7)	Complex span tasks
Executive Control	4	r = 0.5–0.7	Large (d > 0.8)	Interference resolution tasks
Temporal Perception	3	r = 0.6–0.8	NR *	Rhythm discrimination tasks
Cognitive Flexibility	5	r = 0.3–0.5	Moderate (d = 0.4–0.6)	Task-switching paradigms
Anxiety Regulation	3	r = 0.4–0.6	Moderate (d = 0.5–0.7)	Sport-specific anxiety measures

NR * = effect size not reported in extracted data.

**Table 12:** Expertise-level differences in cognitive performance.

Expertise Level	Attention Performance	Working Memory	Temporal Precision	Cognitive Flexibility	Overall Advantage
Elite International	95th percentile	90th percentile	95th percentile	85th percentile	25–30% vs. recreational
National/Regional	80th percentile	75th percentile	80th percentile	70th percentile	15–20% vs. recreational
Club/Competitive	65th percentile	60th percentile	65th percentile	60th percentile	8–12% vs. recreational
Recreational	50th percentile	50th percentile	50th percentile	50th percentile	Baseline reference

**Table 13:** Cognitive intervention effectiveness summary.

Intervention Type	Studies (*n*)	Effect Size Range	Duration	Cognitive Improvements	Performance Improvements
Neurofeedback Training	3	d = 0.8–1.4	8–12 weeks	Attention, executive control	10–25% cognitive performance
Attention Training	3	d = 0.6–1.2	4–8 weeks	Focus regulation, interference resistance	5–15% swimming speed
Psychological Skills	2	d = 0.7–1.1	6–10 weeks	Anxiety reduction, confidence	8–20% performance consistency
Combined Approaches	2	d = 1.0–1.5	10–16 weeks	Multiple domains	15–30% overall improvement

**Table 14:** Intervention implementation guidelines.

Implementation Factor	Neurofeedback	Attention Training	Psychological Skills	Combined Programs
Optimal Frequency	3–4× per week	2–3× per week	2× per week	Variable by component
Session Duration	30–45 min	20–30 min	45–60 min	60–90 min
Training Period	8–12 weeks	4–8 weeks	6–10 weeks	12–16 weeks
Individual vs. Group	Individual preferred	Both effective	Group effective	Individual for elite
Trainer Qualifications	Certified neurofeedback	Sport psychology	Licensed practitioner	Multi-disciplinary team

**Table 15:** Individual difference factors and moderation effects.

Moderating Factor	Studies (*n*)	Effect Size	Primary Domains Affected	Practical Implications
COMT Genotype	2	d = 0.5–0.8	Working memory, stress tolerance	Personalized training stress
Training History	8	d = 0.6–1.2	Neural efficiency, cognitive flexibility	Experience-based programming
Age/Development	6	d = 0.4–0.9	Neuroplasticity, strategic thinking	Age-appropriate interventions
Gender	4	d = 0.3–0.7	Attention strategies, anxiety responses	Gender-specific approaches
Personality Traits	3	d = 0.4–0.8	Intervention responsiveness, burnout	Individualized interventions
Environmental Context	5	d = 0.5–1.0	Skill transfer, adaptation patterns	Context-specific training

**Table 16:** Personalized training recommendations based on individual differences.

Individual Profile	Optimal Cognitive Training	Intervention Approach	Expected Outcomes	Monitoring Strategies
COMT Met/Met, Young	High cognitive load, complex skills	Neurofeedback + attention training	Superior learning, stress sensitivity	Stress monitoring, technique focus
COMT Val/Val, Experienced	Stress simulation, competition preparation	Psychological skills + imagery	Consistent performance under pressure	Performance variability tracking
High Training History	Advanced cognitive strategies	Individual, specialized interventions	Refined cognitive efficiency	Long-term tracking metrics
Early Career, High Openness	Novel, varied cognitive training	Group + individual approaches	Rapid skill acquisition	Breadth of improvement assessment
Masters/Older Athletes	Cognitive maintenance, strategic focus	Flexibility + strategic training	Maintained cognitive performance	Age-appropriate benchmarking

## Data Availability

No new data were created or analyzed in this study.

## Supplementary Materials

The following supporting information can be downloaded at https://www.mdpi.com/article/10.3390/brainsci16010116/s1. Table S1: Abbreviation List; Table S2.1: Quantitative Effect Sizes Across Included Studies; Table S2.2: Summary of Contradictory Findings Across Studies; Table S2.3: Neuroimaging Findings by Brain Region and Modality; Table S2.4: Intervention Study Characteristics and Outcomes; Table S2.5: Sample and Methodological Characteristics Summary; Table S3: PRISMA 2020 Checklist. Ref. [195] was cited in Supplementary Materials.
