# Supplementary material for: Neural Efficiency and Sensorimotor Adaptations in Swimming Athletes: A Systematic Review of Neuroimaging and Cognitive–Behavioral Evidence for Performance and Wellbeing
Source: Brain Sci. 2026 Jan 22;16(1):116. doi: 10.3390/brainsci16010116 (PMC12839007; doi:10.3390/brainsci16010116)
Supplement: Supplementary file 1 [file brainsci-16-00116-s001.zip › Sup_Table_S1_Abbreviation List.pdf]

Table S1. Abbreviation List

Abbreviation	Definition
	**Neuroimaging and Brain Techniques**
DTI	Diffusion Tensor Imaging
EEG	Electroencephalography
ERP	Event-Related Potentials
fMRI	Functional Magnetic Resonance Imaging
MEG	Magnetoencephalography
MRI	Magnetic Resonance Imaging
NIRS	Near-Infrared Spectroscopy
rs-fMRI	Resting-state Functional Magnetic Resonance Imaging
sMRI	Structural Magnetic Resonance Imaging
tDCS	transcranial Direct Current Stimulation
TMS	Transcranial Magnetic Stimulation
	**Neural Signal Processing and Connectivity**
FA	Fractional Anisotropy
FC	Functional Connectivity
Hz	Hertz
MD	Mean Diffusivity
MMN	Mismatch Negativity
PLI	Phase Lag Index
SMR	Sensorimotor Rhythm
SNR	Signal-to-Noise Ratio
	**Brain Regions and Networks**
mPFC	medial Prefrontal Cortex
pSTS	posterior Superior Temporal Sulcus
ROI	Region of Interest
STS	Superior Temporal Sulcus
TPJ	Temporoparietal Junction
	**Statistical and Analytical Methods**
ANOVA	Analysis of Variance
CCA	Canonical Correlation Analysis
DFA	Detrended Fluctuation Analysis
ICA	Independent Component Analysis
LASSO	Least Absolute Shrinkage and Selection Operator
LDA	Linear Discriminant Analysis
mRMR	minimum Redundancy Maximum Relevance
MVPA	Multivariate Pattern Analysis
PCA	Principal Component Analysis
RFE	Recursive Feature Elimination
SD	Standard Deviation
SPSS	Statistical Package for the Social Sciences
	**Artificial Intelligence and Machine Learning**
AI	Artificial Intelligence
CNN	Convolutional Neural Network
CSP	Common Spatial Patterns
DBSCAN	Density-Based Spatial Clustering of Applications with Noise
LSTM	Long Short-Term Memory

ML	Machine Learning
RNN	Recurrent Neural Network
SHAP	Shapley Additive exPlanations
SVM	Support Vector Machine
**Cognitive and Psychological Assessments**	
PMA	Primary Mental Ability
PST	Psychological Skills Training
RPE	Rate of Perceived Exertion
SRS	Social Responsiveness Scale
ToM	Theory of Mind
VAS	Visual Analog Scale
**Genetic and Molecular Biology**	
COMT	Catechol-O-Methyltransferase
DNA	Deoxyribonucleic Acid
PCR	Polymerase Chain Reaction
SNP	Single Nucleotide Polymorphism
**Physiological Measures**	
BMI	Body Mass Index
EMG	Electromyography
HRV	Heart Rate Variability
VEF	Visual Evoked Field
**Swimming and Sports-Specific Terms**	
FINA	Fédération Internationale de Natation
IdC	Index of arm Coordination
