# Supplementary material for: Neural Efficiency and Sensorimotor Adaptations in Swimming Athletes: A Systematic Review of Neuroimaging and Cognitive–Behavioral Evidence for Performance and Wellbeing
Source: Brain Sci. 2026 Jan 22;16(1):116. doi: 10.3390/brainsci16010116 (PMC12839007; doi:10.3390/brainsci16010116)
Supplement: Supplementary file 1 [file brainsci-16-00116-s001.zip › Sup_Table_S2_Quantitative_Synthesis.pdf]

Supplementary Tables S2

Quantitative Synthesis Tables for Swimming Neuroscience Systematic Review

Neural Efficiency and Sensorimotor Adaptations in Swimming Athletes: A Systematic Review of Neuroimaging and Cognitive-Behavioral Evidence for Performance and Wellbeing

Table S2.1: Quantitative Effect Sizes Across Included Studies

Effect sizes extracted or calculated from studies with sufficient quantitative data for swimmer vs. control comparisons.

Study ID	Authors (Year)	N	Comparison	Outcome	Effect Size	95% CI	p-value
159	Pei et al. (2021)	44	Swimmers vs Controls	Reaction time	d = 0.69	[0.07, 1.31]	0.033
159	Pei et al. (2021)	44	Swimmers vs Controls	Accuracy	d = 1.07	[0.43, 1.71]	0.005
159	Pei et al. (2021)	44	Swimmers vs Controls	Upper beta connections	d = 0.76	[0.14, 1.38]	0.040
144	Aly et al. (2019)	22*	Swimmers vs Irregular	P3 Amplitude	d = 1.31	[0.38, 2.24]	<0.05
144	Aly et al. (2019)	22*	Swimmers vs Irregular	P3 Latency	d = 0.82	[-0.05, 1.69]	<0.01
161	Penna et al. (2017)	NR	Mental fatigue effect	Swim time	d = 0.13†	NR	<0.05
161	Penna et al. (2017)	NR	Mental fatigue effect	Mean speed	d = 0.14†	NR	<0.05
151	Huang et al. (2017)	NR	FC-Ranking correlation	World ranking	r^2 = 0.41	NR	<0.001
143	Abe et al. (2017)	57	COMT genotype effect	FINA points	NR	NR	0.026

*Note: d = Cohen's d; r^2 = coefficient of determination; NR = not reported; *Swimmers vs irregular exercisers comparison (total N = 33 across three groups); †Effect sizes reported in original paper. 95% CI calculated using $SE = \sqrt{(2/n)} \times \sqrt{(1 + d^2/8)}$ where sample sizes were available.*

Table S2.2: Summary of Contradictory Findings Across Studies

Contradictions identified between studies and possible explanations for inconsistent findings.

Domain	Finding A	Study A	Finding B	Study B	Possible Explanation
Mental Fatigue	Impairs performance (↓1.2%, $d = 0.13$)	Penna et al. 2017 (adult competitive)	No significant effect ($p = 0.49$)	Penna et al. 2021 (masters)	Age-related resilience; accumulated coping strategies in veteran swimmers
Attentional Focus	External focus improves speed ($p < 0.05$)	Freudenheim et al. 2010 (intermediate)	No difference between conditions	Maloney & Gorman 2021 (expert)	Expertise level differences; task complexity (continuous swimming vs discrete dive start)
Attentional Focus	Internal focus impairs speed ($p < 0.05$)	Stoate & Wulf 2011 (expert)	No difference between conditions	Maloney & Gorman 2021 (expert)	Different tasks (25m swim vs dive start); measurement sensitivity; ecological validity
Cognitive Superiority	Swimmers superior to controls ($d = 0.69$-1.07, $p < 0.05$)	Pei et al. 2021	Non-significant trend only	Yao et al. 2023	Sample size differences ($N = 44$ vs 28); different cognitive tasks assessed
Neural Efficiency Indicators	Sparser connectivity = efficiency (EEG)	Pei et al. 2021	Stronger FC = better ranking (fMRI)	Huang et al. 2017	Different modalities measure different constructs; region-specific effects; phase-lag index ≠ BOLD FC

Note: FC = functional connectivity; These contradictions could not be fully reconciled due to methodological heterogeneity across studies. Apparent inconsistencies may reflect true moderating effects (age, expertise) or methodological differences rather than theoretical contradictions.

Table S2.3: Neuroimaging Findings by Brain Region and Modality

Systematic organization of neuroimaging findings by anatomical region and assessment modality.

Brain Region	Modality	Finding	Direction	Quantitative Effect	Study	Quality
Prefrontal Cortex	ERP	Executive control activation	Enhanced	NR	Doi et al. 2019	Moderate
Thalamus	fMRI	Functional connectivity to sensorimotor	↑ Stronger	$r^2 = 0.41$, $p < 0.001$	Huang et al. 2017	High
Sensorimotor Cortex	fMRI	Thalamic connectivity	↑ Stronger	$r = 0.64$	Huang et al. 2017	High
Motor Cortex	TMS	Intracortical inhibition in water	↑ Increased	NR	Sato et al. 2020	Moderate
Parietal Cortex	EEG	Upper beta connectivity	↓ 35% sparser	$d = 0.76$, $p = 0.040$	Pei et al. 2021	High
Parietal Cortex	EEG	Lateralization index	↑ Higher	$p = 0.012$	Pei et al. 2021	High
Frontal Lobe	EEG	Spectral amplitude modulation	Modulated	NS	Mikicin 2022	Low
Occipital Lobe	EEG	Alpha rhythm intensity	↑ Higher	$p \le 0.05$	Ivanyuk et al. 2023	Moderate
Whole Brain	EEG	Alpha rhythm (all regions)	↑ Enhanced	$p \le 0.01$	Ivanyuk et al. 2023	Moderate
Pz electrode site	ERP	P3 amplitude	↑ Enhanced	$d = 1.31$, $p < 0.05$	Aly et al. 2019	Moderate
Pz electrode site	ERP	P3 latency	↓ Shorter	$d = 0.82$, $p < 0.01$	Aly et al. 2019	Moderate
Multiple Regions	fMRI	Working memory and action inhibition task activation	Altered patterns	NS (trend)	Yao et al. 2023	Moderate
Multiple Regions	EEG	Neurofeedback-EEG during physical exercise	↑ Improved	$p < 0.05$	Mikicin et al. 2020	Low

Note: ↑ = increased/enhanced; ↓ = decreased/reduced; NS = not significant; NR = not reported. Quality ratings based on risk-of-bias assessment.

Table S2.4: Intervention Study Characteristics and Outcomes

Summary of intervention studies examining effects on swimming performance and neural outcomes.

Study	Intervention Type	Duration	N	Control	Primary Outcome	Effect	p-value	Quality
Sheard & Golby 2006	PST (goal-setting, visualization, relaxation)	7 weeks, 45 min/wk	NR	None	200m stroke times	↓ Improved (3/13 events)	<0.05	Low
Mikicin et al. 2020	Neurofeedback-EEG + exercise	20 sessions	NR	None	Mental work performance	↑ Improved	<0.05	Low
Mikicin 2022	Neurofeedback-EEG training	Multiple sessions	10	None	EEG spectral amplitude	Changed	NS	Low
Couture et al. 1999	Associative attention strategy	Single session	69	Yes	Swim time	↓ Faster	<0.05	Moderate
Freudenheim et al. 2010	External focus instructions	Single session	NR	Yes	Swim time	↓ Faster	<0.05	Moderate
Stoate & Wulf 2011	External focus instructions	Single session	NR	Yes	Swim speed	No diff vs control	NS	Moderate
Penna et al. 2021	tDCS brain stimulation	Single session	10	Sham	800m swim time	No effect	0.62	Moderate
Maloney & Gorman 2021	External/internal focus instructions	Single session	NR	Yes	Dive start metrics	No diff	NS	Moderate

Note: PST = Psychological Skills Training; tDCS = transcranial direct current stimulation; NS = not significant; NR = not reported. Quality ratings: Low = no control group; Moderate = control group present. Of 8 intervention studies, 4 demonstrated significant effects and 4 showed null effects.

Table S2.5: Sample and Methodological Characteristics Summary

Overview of participant and methodological characteristics across 24 included studies.

Characteristic	Value	Details / Notes
Total included studies	24	Systematic review final inclusion
Total unique participants	~550	Estimated across all studies (some potential sample overlap)
Median sample size	36	Range: 10-69 (verified from 11 studies with clearly reported N)
Studies with N < 30	5/24 (21%)	Underpowered for detecting moderate effects
Studies with N ≥ 50	3/24 (13%)	Couture 1999 (N=69), Abe 2017 (N=57), Parnabas 2015 (N=69)
Cross-sectional design	21/24 (88%)	Precludes causal inference about training effects
Intervention/experimental studies	9/24 (38%)	Variable quality; 3 without control groups
Studies with control group	15/24 (63%)	Non-athlete or recreational comparison groups
Male only samples	2/24 (8%)	Limited generalizability to female athletes
Female only samples	1/24 (4%)	Bekendam et al. 2019
Mixed gender samples	21/24 (88%)	Sex-specific analyses rarely conducted
Elite/International level	9/24 (38%)	National team or international competitors
Neuroimaging studies	9/24 (38%)	EEG: 4, fMRI: 2, TMS: 1, ERP: 2
Behavioral-only studies	15/24 (63%)	Cognitive performance without neural assessment
Participant age range	17-adult masters	Across all included studies
Required N for 80% power	64 per group	For d = 0.5, α = 0.05, two-tailed test
Studies meeting power threshold	0/24 (0%)	ALL studies underpowered for moderate effects
Publication year range	1999-2023	67% published 2017-2023
Studies from 2019-2024	9/24 (38%)	Contemporary evidence base

Note: Power calculation assumes medium effect size (d = 0.5) based on observed effects in this review. The median sample size of 36 provides only ~50% power to detect medium effects, substantially below the conventional 80% threshold. These pervasive methodological limitations constrain confidence in synthesized findings and highlight the critical need for larger, longitudinal investigations.
